# Self-assembled traditional Chinese nanomedicine modulating tumor immunosuppressive microenvironment for colorectal cancer immunotherapy

**DOI:** 10.7150/thno.72509

**Published:** 2022-08-15

**Authors:** Qianqian Mao, Juan Min, Rui Zeng, Haiqin Liu, Hao Li, Cao Zhang, Aixian Zheng, Jiumao Lin, Xiaolong Liu, Ming Wu

**Affiliations:** 1 The United Innovation of Mengchao Hepatobiliary Technology Key Laboratory of Fujian Province, Mengchao Hepatobiliary Hospital of Fujian Medical University, Fuzhou 350025, P. R. China.; 2 Academy of Integrative Medicine, Fujian University of Traditional Chinese Medicine, Fuzhou 350122, P.R. China.; 3 Wuhan Institute of Virology, Chinese Academy of Sciences, Wuhan 430071, P. R. China.; 4 Mengchao Med-X Center, Fuzhou University, Fuzhou 350116, P. R. China.; 5 Fujian Key Laboratory of Integrative Medicine on Geriatrics, Fujian University of Traditional Chinese Medicine, Fuzhou, Fujian 350122, P.R. China.

**Keywords:** lentinan, ursolic acid, traditional Chinese nanomedicine, colorectal cancer, immunotherapy

## Abstract

Colorectal cancer (CRC), mostly categorized as a low immunogenic microsatellite-stable phenotype bearing complex immunosuppressive tumor microenvironment (TME), is highly resistant to immunotherapy. Seeking safe and efficient alternatives aimed at modulating tumor immunosuppressive TME to improve outcome of CRC is highly anticipated yet remains challenging.

**Methods:** Enlightened from the drug complementary art in traditional Chinese medicine, we designed a self-assembled nanomedicine (termed LNT-UA) by the natural active ingredients of ursolic acid (UA) and lentinan (LNT) through a simple nano-precipitation method, without any extra carriers, for CRC immunotherapy.

**Results:** UA induces immunogenic cell death (ICD), while LNT further promotes dendritic cell (DC) maturation and repolarizes tumor-associated macrophage (TAM) from a protumorigenic M2 to an antitumor M1 phenotype. Co-delivery of UA and LNT by LNT-UA effectively reshapes the immunosuppressive TME and mobilizes innate and adaptive immunity to inhibit tumor progression in the CT26 CRC tumor model. Following the principle of integrative theoretical system of traditional Chinese medicine (TCM) on overall regulation, the further combination of LNT-UA and anti-CD47 antibody (αCD47) would reinforce the antitumor immunity by promoting phagocytosis of dying tumor cells and tumor-associated antigens (TAAs), leading to effective suppression of both primary and distant tumor growth with 2.2-fold longer of median survival time in the bilateral tumor model. Most notably, this combination effect is also observed in the spontaneous CRC model induced by chemical carcinogens, with much less and smaller size of tumor nodules after sequential administration of LNT-UA and αCD47 through gavage and intraperitoneal injection, respectively.

**Conclusions:** This study provides a promising self-assembled traditional Chinese nanomedicine to improve immunotherapy for CRC, which might be applicable for future clinical translation.

## Introduction

Colorectal cancer (CRC) is one of the most frequent malignant tumors and the major causes of cancer related death worldwide, accounting for 10% of new cancer cases and deaths 1. Immunotherapy shows a tremendous promise of treatment in several cancer types but is much less effective on colorectal cancer with only 5-15% of CRC patients featured as microsatellite instability or mismatch repair deficiency which are benefiting from current immune checkpoint blockade (ICB), while more than 90% of the patient population fails to respond to ICB 2, 3. In addition, different from many other organs, the colon is persistently exposure to intestinal nutrients- and microflora- related non-self-antigens, leading to its immune system generating a more complex immunosuppressive microenvironment with low immunogenicity and the lack of immune cell infiltration 4. Recent studies suggest that some chemotherapy drugs (*e.g.,* mitoxantrone, oxaliplatin) not only have cytotoxicity against tumor cells, but also can induce immunogenic cell death (ICD) to elicit the anti-tumor immune response as an abscopal effect through releasing tumor-associated antigens (TAAs), damage-associated molecular patterns (DAMPs) and other immune stimulatory factors 5, 6. However, the resulting immunogenicity is still not sufficient to boost antitumor immunity to efficiently prohibit tumor progression, recurrence and metastasis. In addition, conventional cytotoxic chemotherapeutic drugs also have the disadvantages of poor efficacy, low bioavailability, drug resistance and myelosuppression 7. Therefore, seeking safe and efficient alternatives aimed at triggering ICD and eliciting robust immunity to improve outcome of CRC is highly anticipated yet remains challenging.

In recent years, growing attentions have been driven toward seeking traditional Chinese medicines (TCMs) as potential modulators of tumor immunotherapy 8. Especially, the combinational spirit and integrative theoretical system of TCM in the Chinese people's experience, such as “strengthening the body (*Fu Zheng*) or eliminating evil (*Qu Xie*)” with a long history, are highly consistent with the mechanism of immunotherapy by restoring the patient's immune system to eliminate cancer cells. Indeed, numerous empirical prescriptions, monomers and extracts of TCM have been used in modulating systemic immunity, whether implemented alone or incorporated with western modern therapies 9, 10. Among them, ursolic acid (UA) as a pentacyclic triterpenoid compound derived from *Prunella vulgaris* L., *Hedyotis diffusa* Willd. or *Plantago asiatica* L, has been regarded as a highly effective lead compound for immune-modulation through affecting the JAK/STAT signaling pathway in lymphocytes 11. In addition, it is also capable of inducing tumor cell apoptosis by imposing endoplasmic reticulum (ER) stress related to ASK1-JNK signaling pathway or modulating the protein expression of Caspase and Bcl-2 family 12-14, with the merits of multiple biologic activities, low toxicity and broad sources in comparison with conventional cytotoxic chemical drugs 11, 15, 16. These pathways are closely related to the generation of ICD effects, so we speculate that UA may induce ICD of tumor cells; but UA has poor water solubility, leading to the low bioavailability *in vivo* 17. Despite the emergence of nanotechnology providing a novel strategy for UA delivery, this strategy still fails to fully expand the application of UA due to the limited drug loading capacity, inevitable side effects raised from extra excipients, and complicated synthetic methods 18, 19. One promising strategy is to develop carrier-free nanodrug (CFND) fabricated only from the pure active drugs without the assistance of any additional carriers 20-22, but how to seek and align therapeutic agents with different structures and physicochemical features into “all-in-one” CFND for the sake of exerting certain collaborative functions remains an unmet challenge. As far as we know, CFND self-assembled from TCM for combinational CRC immunotherapy has been rarely reported.

Lentinan (LNT), as a kind of water-soluble polysaccharide consisting of β-(1→3)-glucan backbone with β-(1→6)-glucose branch chain extracted from Lentinus edodes (Berk.) sing, is frequently used in clinic as a host defense potentiator 23, 24. Recent studies report that LNT can promote the antigen processing and presentation by recognizing specific pattern recognition receptors (PRRs), such as toll-like receptors (TLRs) and Dectin-1 on antigen presenting cells (APCs), activate the macrophage phagocytosis through nuclear factor kappa-B (NF-κB) and TLR signaling pathways, and induce antitumor-relative cytokine release to exert immunomodulatory effects 25, 26. Moreover, LNT is also a natural polymer material, which is characterized by high stability, hydrophilicity, good biocompatibility and biodegradability 27. Therefore, LNT might be simultaneously served as a biocompatible excipient to improve the solubility and bioavailability of UA, as well as a versatile immunopotentiator to work in synergy with UA.

**Scheme 1:**
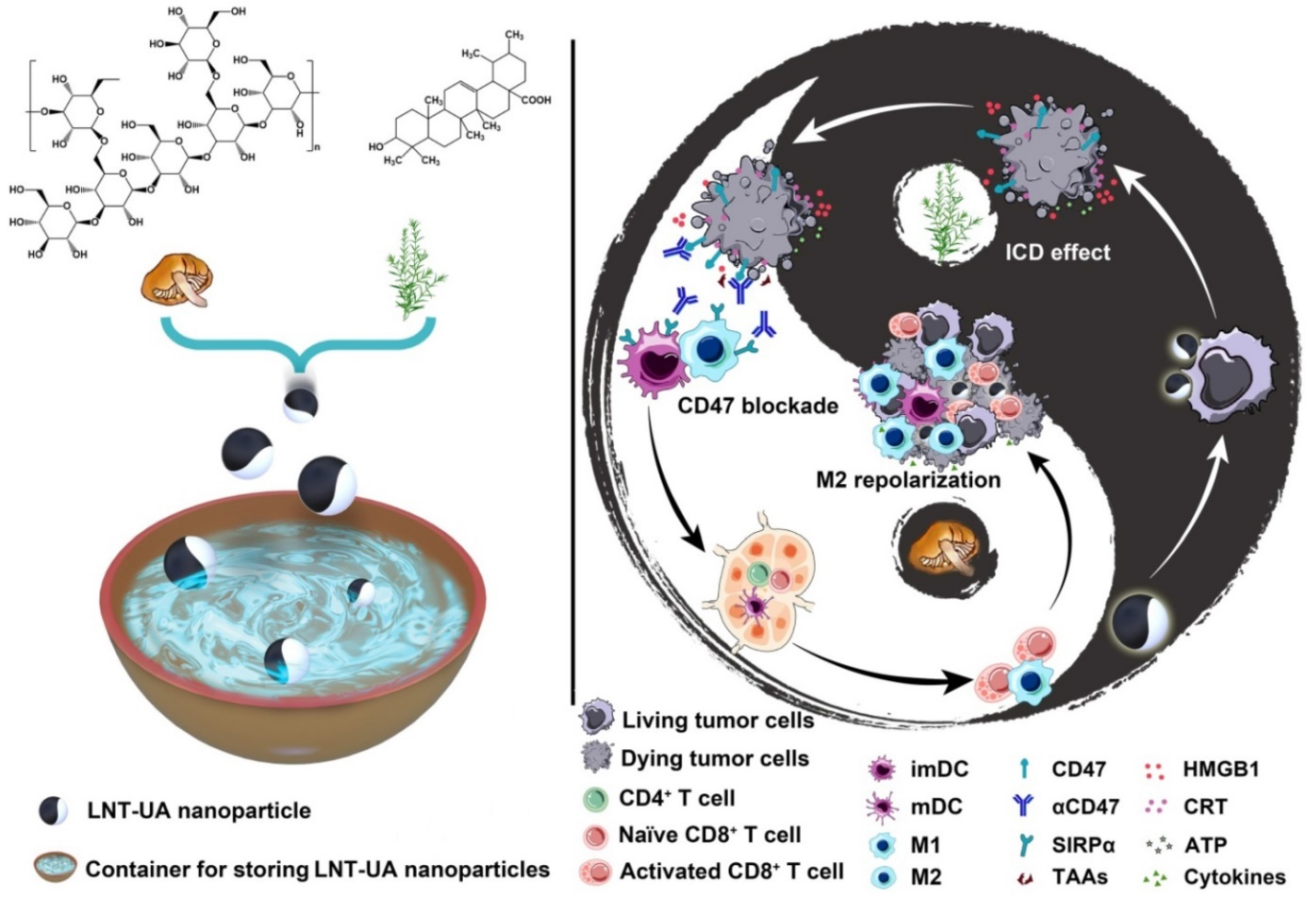
** Schematic illustration of LNT-UA preparation and combination with αCD47 for CRC immunotherapy by modulating tumor immunosuppressive microenvironment.** The ball represents LNT-UA nanoparticle, where the black part indicates UA element to induce immunogenic cancer cell death (ICD), while LNT labeled with white color acts as an adjuvant to promote dendritic cell (DC) maturation and repolarizes tumor-associated macrophage (TAM) from a protumorigenic M2 to an antitumor M1 phenotype. The bowl represents the container for storing LNT-UA nanoparticles. Co-delivery of UA and LNT by LNT-UA effectively reshapes the immunosuppressive TME and mobilizes innate and adaptive immunity to inhibit tumor progression in the CT26 CRC tumor model.

Even if there are sufficient TAAs and reprogrammed phagocytes induced by the incorporation role of LNT and UA in the tumor microenvironment (TME), one mechanism that might diminish LNT-UA efficiency is the persistence of a large number of antiphagocytic molecules like CD47, which will send a “don't eat me” signal to inhibit phagocytosis and sequentially attenuate the tumor antigen presentation to T cells *via* binding to the signal regulatory protein alpha (SIRPα) receptor 28. In this context, it would be ideal for amplifying immune response in CRC by the combination of LNT-UA based TCM and CD47 targeted ICB. Based on these surveys, we designed a stable nanodrug (LNT-UA) self-assembly from LNT and UA without additional carriers using a nanoprecipitation method in this study (Scheme 1). LNT-UA induced apoptosis and activated immunogenic cell death in CRC cells more effectively in comparison with free UA. We also found that LNT-UA had remarkable antitumor effects in mouse CRC tumor models, and the mechanism was related to immunogenic cell death and its subsequent activation of antitumor immunity. Furthermore, the combination of LNT-UA and anti-CD47 antibody (αCD47) at tumor site achieved the abscopal effect in mouse bilateral CRC model, which would ultimately prolong survival of the host. Meanwhile, their combination effect is also proved in spontaneous CRC model induced by chemical carcinogens. Thereby, this modernized natural product formulation (*Fufang*, LNT-UA) based on TCM theory focusing on the overall regulation offers a promising strategy for CRC immunotherapy.

## Materials and Methods

### Materials

Lentinan (DX0085) was obtained from Desite^@^ (Chengdu, China). Ursolic acid (A1810063) was obtained from Aladdin^@^ (Shanghai, China). BCA Protein Assay Kit (DQ111-01) and Cell Counting Kit-8 (CCK-8, FC101-02) were purchased from TransGen Biotech Co., Ltd. (Beijing, China). Live/Dead Cell Staining Kit (C542) and Annexin V-FITC/PI Apoptosis Detection (AD10) were obtained from Dojindo (JPN). Primary antibodies were purchased from Abcam (USA). Fluorescent secondary antibodies and streaming antibodies were purchased from Thermo Fisher Scientific Inc. (USA). Adenosine triphosphate (ATP) assay kit (S0026) and TdT-mediated dUTP Nick-End Labeling (TUNEL) assay kit (C1088) were obtained from Beyotime Biotech Co., Ltd. (Shanghai, China). *In vivo* MAb anti-mouse CD47 (BE0270) was obtained from Bio X Cell (USA). Azoxymethane (AOM, A5486) was purchased from Sigma (USA). Dextran sulfate sodium (DSS, 216011090) was obtained from MP Biomedicals (USA). Granulocyte macrophage colony-stimulating factors (GM-CSF, 415-ML-020/CF) and interleukin-4 (IL-4, 404-ML-010/CF) were purchased from R&D Systems (USA). Enzyme-Linked Immunosorbent Assay (ELISA) kits were obtained from Boster Biological Technology Co., Ltd. (Wuhan, China). All other chemicals were used as obtained unless otherwise specified.

### Preparation and characterization of LNT-UA

LNT-UA was prepared by a nanoprecipitation method. Briefly, LNT (10 mg) and UA (5 mg) were co-dissolved in 1 mL of dimethyl sulfoxide (DMSO), followed by dropwise addition into 2 mL of ultrapure water under continuously stirring. Subsequently, the crude product was dialyzed against ultrapure H_2_O with a dialysis bag of molecular weight cut-off (MWCO) of 8000-14000 for 72 h to remove the organic solvent and unloaded the free drug.

The morphology and particle diameter of LNT-UA nanoparticles were observed using a transmission electron microscope (TEM) and scanning electron microscopy (SEM). For TEM, LNT-UA was negatively stained with 1% uranium acetate on a copper grid. The hydrodynamic diameters and zeta potential of LNT-UA were measured by a Zetasizer Nano ZS (Malvern Instruments, Southborough, MA). The chemical structures of LNT-UA were analyzed by a Fourier transform infrared (FT-IR) spectrometer (Perkin-Elmer, Spectrum-2000). The encapsulation efficiency and loading capacity of LNT-UA were analyzed by the high-performance liquid chromatography (HPLC) system (Agilent Technologies, 1260 LC), as previously reported 29. The drug encapsulation efficiency was defined as (entrapped UA weight/feeding UA weight) × 100%. Loading capacity was calculated by (entrapped UA weight/total LNT-UA weight) × 100%.

### *In vitro* drug release

The release profiles of LNT and UA from LNT-UA were investigated using the dialysis method in different buffers (pH 5.0, 6.5 or 7.4) containing 0.1% (v/v) Tween 80. Briefly, 0.8 mL of LNT-UA was sealed in dialysis bags (MWCO 8000-14000) with 7.2 mL of different buffers outside and incubated at 37 °C and stirred at 100 rpm. Samples were withdrawn at predetermined time intervals, and the LNT and UA content of each sample were detected by phenol-sulfuric acid method and HPLC, respectively.

### Cell uptake analysis

5×10^6^ murine colon tumor cell CT26 cells were seeded in a 10 cm culture plate. After 24 h of incubation, the medium was replaced by the fresh one containing free UA or LNT-UA (UA: 22 μmol/L) for 4 or 8 h. As for free UA formulation, it was firstly pre-dissolved in DMSO and then diluted in culture medium. After treatments, a part of cell suspension was lysed by radio-immunoprecipitation assay (RIPA) lysis buffer for determining total protein content by a BCA Protein Assay Kit. The rest of cell suspension was frozen and thawed repeatedly 3 times, followed by the addition of the same volume of methanol to extract UA. The UA concentration was determined by using HPLC. Finally, the cell uptake was quantitatively analyzed by normalizing UA content to the corresponding total protein weight.

### *In vitro* antitumor effect

Cell viabilities were determined by CCK-8, Live/Dead staining as well as Annexin V-FITC/PI assay. For the CCK-8 assay, 1.5×10^4^ cells per well were seeded in a 96-well plate and incubated for 24 h. Then, cells were incubated with the fresh medium containing various concentrations of free UA, LNT, or LNT-UA for 24 h. After removing the supernatant, 10 μL of CCK-8 solution was added to each well followed by incubation for another 1.5 h. Finally, the absorbance was measured using the Spectra Max M5 microplate reader (Molecular Devices, USA) at 450 nm. With respect to the Live/Dead staining assay, the cells after treatments were stained with both calcein AM and propidium iodide (PI) in a 37 °C incubator for 15 min, and then observed and photographed under a fluorescence microscope (Zeiss Axio Vert.A1, Germany). For the Annexin V-FITC/PI assay, Annexin V-FITC and PI were added into the treated cells for staining of 15 min. Finally, the cells were filtered through a 40 μm cell filter before being tested by flow cytometry (BD, FACSVerse, USA).

### Induction of ICD

To verify LNT-UA-induced ICD, surface expression of calreticulin (CRT), extracellular release of high mobility group protein 1 (HMGB1), and ATP secretion were determined *in vitro*. For CRT analysis, CT26 cells were seeded into 35 mm confocal dishes overnight and then incubated with LNT, UA, and LNT-UA at the equivalent UA concentration of 22 μmol/L UA for 4 h. Afterward, the cells were fixed using 4% paraformaldehyde for 15 min and then sealed with 3% BSA in PBS for 30 min. Subsequently, the cells were incubated with anti-CRT antibody (1:500, ab92516) overnight at 4 °C. Afterward, the cells were washed with PBS and stained with anti-rabbit IgG (A-11034, Invitrogen) and DAPI for 1 h and 10 min, respectively. Finally, the cells were observed by a confocal laser scanning microscope (CLSM, Zeiss LSM780). Intracellular HMGB1 distribution was also monitored by the same immunofluorescence analysis. After 8 h of incubation with LNT, UA or LNT-UA, the cells were fixed, permeabilized using 0.3% Triton X-100 and incubated with anti-HMGB1 antibody (1:1000, ab18256) for CLSM observation. The cell surface exposure of CRT and the nucleus level of HMGB1 were also quantitatively analyzed by flow cytometry (BD, FACSVerse, USA). Intracellular ATP level was measured by ATP assay kit, and the cells with the aforementioned treatments were collected and lysed for detection according to the manufacturer's instruction. ATP-induced chemiluminescence was measured by a Spectra Max M5 microplate reader (Molecular Devices, USA).

### DC maturation *in vitro*

Bone marrow-derived dendritic cells (BMDCs) were prepared according to the previously reported method 30, and their maturation after various treatments was checked by determining the expressions of co-stimulatory molecules CD80 and CD86. In brief, the BMDCs were counted and seeded in a 24-well plate. After treatment with various formulations, the cells were harvested, washed by PBS, and then stained with the antibody of CD11c-APC (17-0114-82, eBioscience™), CD80-PE (12-0801-82, eBioscience™), and CD86-PE-Cy7 (25-0862-82, eBioscience™) for flow cytometry analysis. The cells treated with LPS (1 μg/mL) were chosen as the positive control.

### Macrophage polarization *in vitro*

To prepare bone marrow-derived macrophages (BMDMs), bone marrow cells obtained from femurs and tibias were cultured on Dulbecco's modified eagle medium (DMEM) complete medium containing recombinant mouse GM-CSF (20 ng/mL) for 7 days. BMDMs were isolated and incubated on a 24-well plate (5×10^5^/per well). Then, the cells were stimulated by LNT or LPS for 48 h at 37 °C. Then, the cells were stained with anti-CD11b-APC (17-0114-82, eBioscience™) and anti-CD80-PE (12-0801-82, eBioscience™) and then detected by flow cytometry.

### LNT-UA for CRC treatment

Six-week-old male BALB/c mice were ordered from Shanghai China Wushi, Inc. (Shanghai, China). Animal experiment protocols were approved by the Animal Ethics Committee of Mengchao Hepatobiliary Hospital of Fujian Medical University and carried out according to the institutional guidelines.

To establish the animal tumor model, 5×10^5^ CT26 cells in 100 µL of PBS were subcutaneously injected into the right side of the back of BALB/c mice. Afterward, these CT26 tumor-bearing mice were randomized into four groups with the tumor volume of about 100 mm^3^. The mice were then treated with saline (control), LNT, UA, or LNT-UA at the equivalent UA dose of 5 mg/kg through intratumoral injection every three days for three times in total. After the 1^st^ administration, the tumor sizes and body weight were monitored every two days. The tumor size was determined as follows: width^2^×length/2. The tumors of mice were harvested for hematoxylin-eosin (H&E) staining, immunohistochemistry of Ki-67, and immunofluorescence staining for CRT, HMGB1.

### Antitumor activity in bilateral CRC model

To establish CT26 tumor model with primary and distant tumors, 100 µL and 50 µL of cell suspension (5×10^6^/mL) were successively injected into the right (primary tumor) and left (distant tumor) flank of BALB/c mice. Afterward, these CT26 tumor-bearing mice were randomized into four groups with the primary tumor volume of about 100 mm^3^: Control (saline), LNT-UA, αCD47, LNT-UA + αCD47. LNT-UA (UA: 5 mg/kg) was injected intratumorally into the primary tumors on days 1, 5 and 9. The αCD47 antibody (2.5 mg/kg) was injected into the primary tumors intratumorally on days 2, 6 and 10. Body weight and tumor volume were measured every other day, and survival time was also recorded. Animals were euthanized in the presence of impaired health or the size of their tumors exceeded 1500 mm^3^. The bilateral tumors from different groups of mice were harvested for further study, like immunofluorescence staining for CD47 or CD8, H&E, immunohistochemistry of Ki-67, and TUNEL assay for histopathological analysis.

### Antitumor performance in spontaneous CRC model

To establish the spontaneous CRC model, BALB/c mice were randomly divided into five groups: Normal, Control, αCD47, LNT-UA, and LNT-UA+αCD47. The normal group acted as an untreated control. The mice of other groups were intraperitoneally injected with 10 mg/kg of azoxymethane, followed by 7 days of regular diet and water. Mice were then administered with 2% dextran sulfate sodium in drinking water for 6 days and returned to 14 days of regular diet and water until the next cycle of repeated administration of DSS. When the three-time cycle was finished, the mice were treated with different formulations. Typically, LNT-UA (UA: 150 mg/kg) was administrated intragastrically on days 68, 72 and 76, while αCD47 antibody (5 mg/kg) intraperitoneally injected on days 69, 73 and 77. On day 84, the major organs (heart, liver, spleen, lung, kidney) and the whole blood were collected for H&E staining and blood chemistry examination for biosecurity assay. The colon ending invaded with tumor nodules were harvested from different groups of mice for photograph and histopathological observation.

### Dendritic cell (DC) maturation in tumor-draining lymph nodes (TDLNs)

To analyze DC maturation *in vivo*, the TDLNs from CT26-tumor bearing mice were harvested, ground gently and strained by 40 μm filter net to get cell suspension. Then the cells were stained with anti-CD11c-APC, anti-CD80-PE and anti-CD86-PE-Cy7 antibodies for detecting DC maturation by flow cytometry.

### Intratumoral infiltration of immune cells

To analyze tumor infiltrated immune cells, the tumor tissues were harvested post treatment and cut into small pieces, digested with collagenase IV, hyaluronidase, and DNase. The single-cell suspension was further purified by a density gradient centrifugation (Ficoll solution). Then the cells were stained with T lymphocyte markers (anti-CD3-APC, anti-CD4-FITC, and anti-CD8-PE) and macrophage markers (anti-CD11b-APC, anti-CD80-PE, and anti-CD206-PE) before examined by flow cytometry.

### Intracellular cytokine assay

To determine the intratumor levels of interleukin-10 (IL-10), interferon-γ (IFN-γ) and tumor necrosis factor-α (TNF-α), the tumor tissues were homogenized with 200 μL PBS and centrifuged at 14,000×g for 5 min. The cytokines of supernatant were measured by ELISA kits.

### Statistical analysis

Results were shown in this study as mean ± standard deviation (SD). Data between two groups were assessed using independent Student's *t* test (two-tailed). Data in multiple groups were evaluated using the one-way analysis of variance (ANOVA) followed by the *Tukey* post hoc test. A log-rank test was applied for the comparison in the survival study. In addition, the Kruskal-Wallis *H* test was used for the comparison of non-normal distributed data. In this study, *P* < 0.05 was deemed to indicate statistically significant.

## Results and Discussion

### Preparation of LNT-UA

**Figure 1:**
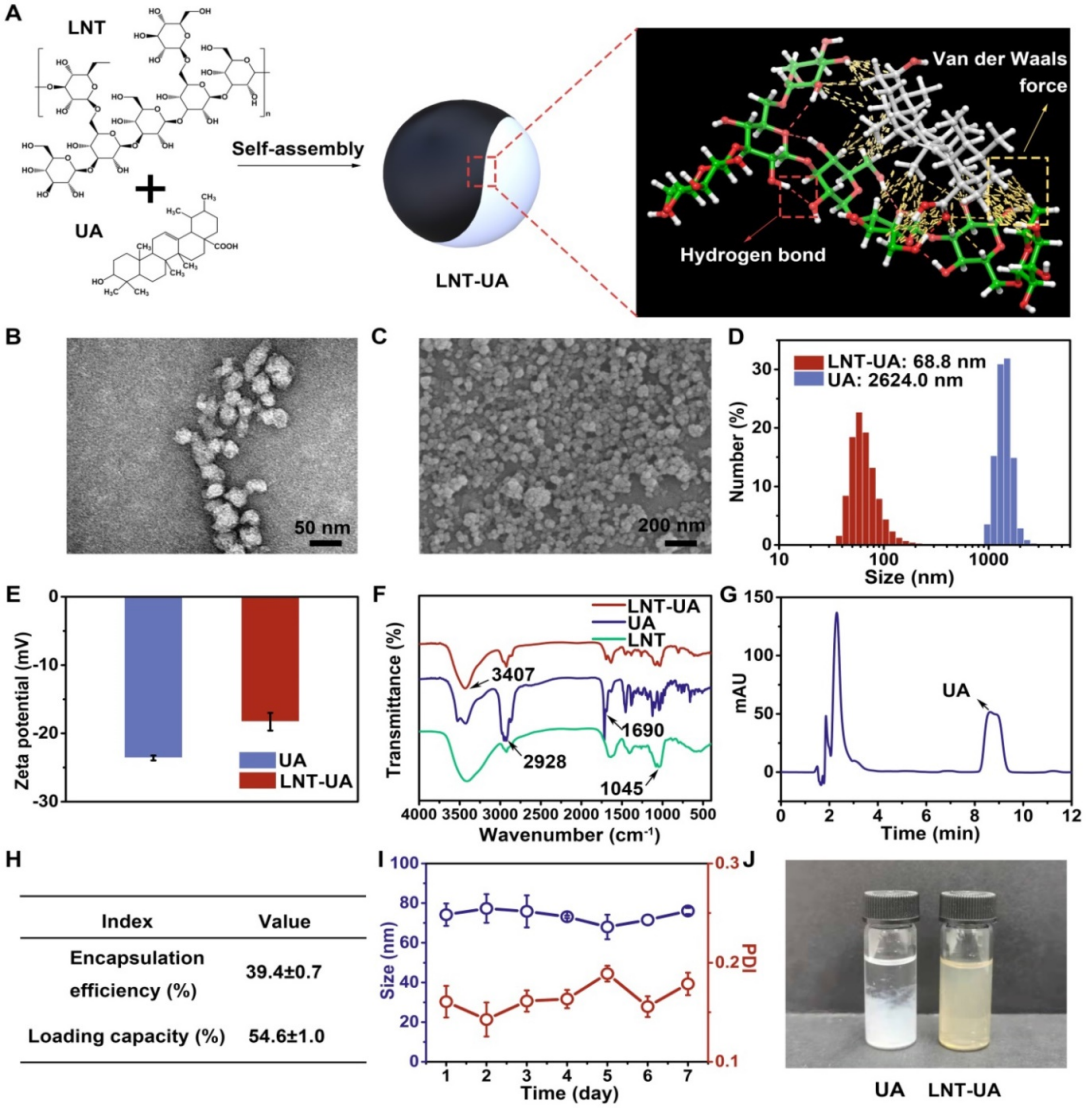
** Preparation and characterization of LNT-UA. (A)** Illustration of interaction forces between LNT and UA for co-self-assembly. **(B, C)** Representative images of LNT-UA by TEM (B) and SEM (C). **(D)** Hydrodynamic size distribution of LNT-UA by DLS. **(E)** Zeta potential of LNT and LNT-UA by DLS. Data are presented as mean ± S.D (n = 3**). (F)** Fourier transform infrared (FT-IR) spectra of LNT-UA, UA and LNT. **(G)** HPLC chromatograms of UA in LNT-UA. **(H)** The encapsulation efficiency and loading capacity of UA in LNT-UA. Data are presented as mean ± S.D (n = 3). **(I)** Particle size and PDI of LNT-UA in PBS for 7 days. Data are presented as mean ± S.D (n = 3). **(J)** Appearance of UA and LNT-UA in PBS stored for 24 h.

The LNT-UA nanodrugs were synthesized by the self-assembly of LNT and UA through the multiple nonbonded interactions such as hydrogen bond and van der Waals force (Figure 1A), and obtained at the optimized feed ratio of 2:1 (W/W) by nanoprecipitation method 31. The morphology of yielded formulations was observed by TEM and SEM. LNT-UA displayed a spherical morphology with an average diameter around 50 nm (Figure 1B-C). The hydrodynamic size of LNT-UA in aqueous solution was further determined by dynamic light scattering (DLS) (Figure 1D). The particle diameter was 68.8 nm with polydispersityindex (PDI) of 0.177, indicating a good dispersity within aqueous environment. The zeta potential of LNT-UA was -18.3 mV (Figure 1E), conferring them to maintain good stability in physiological conditions. In contrast, free UA showed a much larger size of 2624.0 nm (Figure 1D), while its zeta potential was determined to be -23.7 mV (Figure 1E). Moreover, to determine the critical concentration of assembly, the hydrodynamic sizes of LNT-UA at different concentrations were explored. As shown in Figure S1, the hydrodynamic size of LNT-UA maintained constant at the UA concentration above 5.74 μmol/L, and then gradually increased probably due to the aggregation of UA after the separation of LNT from UA at lower concentrations. The chemical component of LNT-UA was confirmed by FT-IR. As shown in Figure 1F, the stretching vibration of O—H at 3407 cm^-1^, C—H at 2928 cm^-1^ and C=O at 1690 cm^-1^ appeared both in the spectra of UA and LNT-UA. In addition, the characteristic peak of 1045 cm^-1^ ascribing to the stretching vibration of glucosidic bond (C—OH and C—O—C) in LNT also appeared in the spectrum of LNT-UA 32. These results demonstrated the successful formation of hybrid nanodrug self-assembly from LNT and UA. HPLC was used to determine the loading capacity and encapsulation efficacy of UA in LNT-UA. As recorded in Figure 1G, the retention time of UA in the chromatogram was approximately 8.4 min. From the results of HPLC, the encapsulation efficacy of UA was 39.4±0.7%, and the loading capacity was 54.6±1.0% (Figure 1H and Figure S2). The particle size of LNT-UA was unchanged significantly in PBS and culture medium supplemented with 10% of FBS (vol/vol) within 7 days, showing that the LNT-UA nanodrugs had good solubility and remarkable stability (Figure 1I, Figure S3 and Table S1). In contrast, the UA precipitated quickly after 24 h, although DMSO and ultrasonic were assisted to pre-dissolve them (Figure 1J). Moreover, the release profiles of LNT and UA from LNT-UA at different pH were analyzed by the phenol-sulfuric acid method and HPLC, respectively. As shown in Figure S4, a much faster and higher LNT and UA release were detected at a citrate phosphate buffer solution of pH 5.0 to mimic the lysosomal environment, with about 54.8% and 63.7% of LNT and UA was respectively released from LNT-UA during 3 d at pH 5.0, whereas the release rates at PBS solution with pH 7.4 (to mimic normal physiological environment) and pH 6.5 (to mimic the weak acidic tumor microenvironment) were not significant.

### LNT-UA eliminates cancer cells

**Figure 2:**
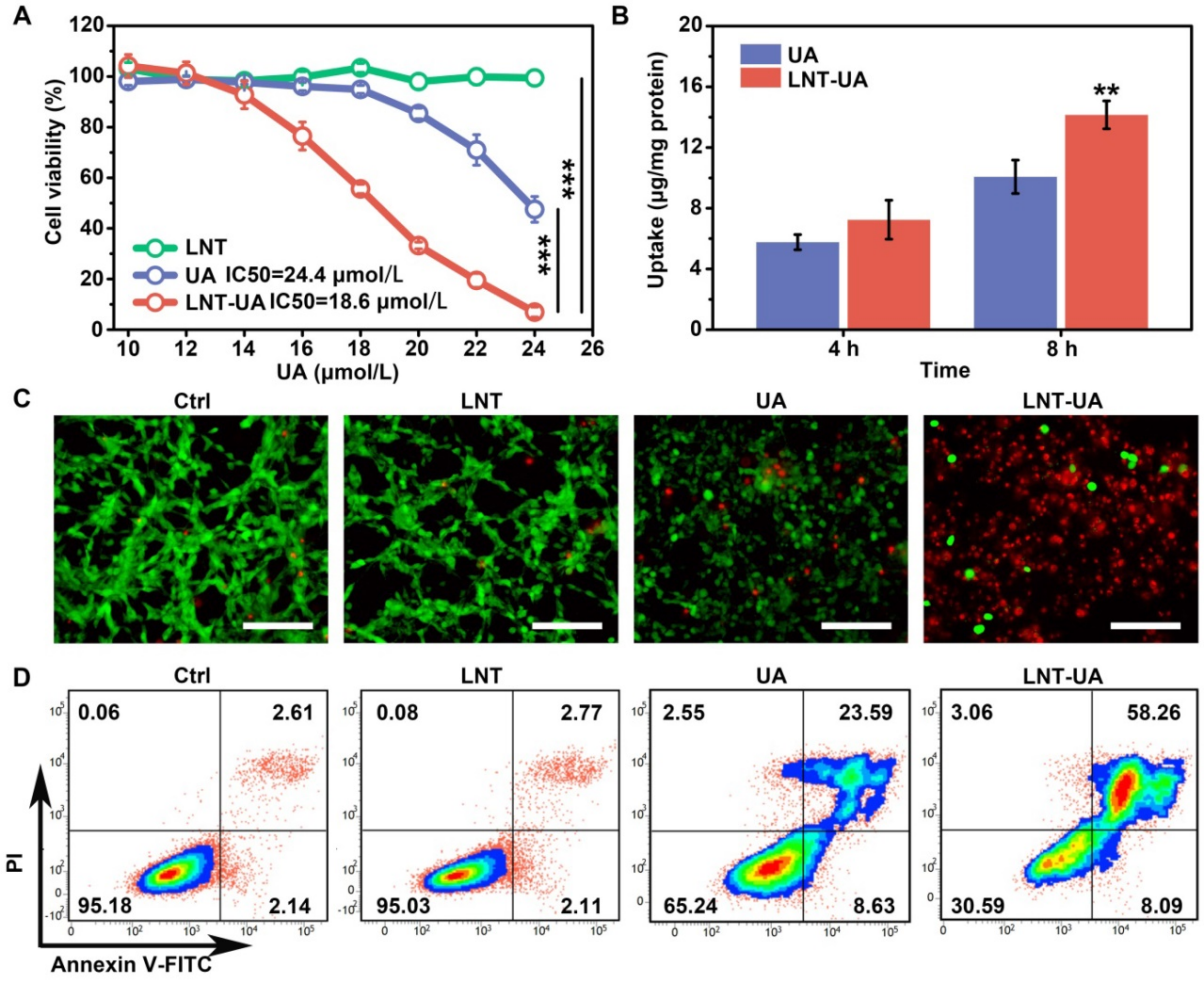
** LNT-UA inhibits tumor cell growth *in vitro*. (A)** Cell viability of CT26 cells treated with LNT, UA, or LNT-UA at different concentrations for 24 h. Data are presented as mean ± S.D (n = 4). ***P < 0.001. **(B)** Uptake of free UA or LNT-UA by CT26 cells for 4 or 8 h. Data are presented as mean ± S.D (n = 3). **P < 0.01. **(C)** Representative fluorescent images of Live/Dead cell staining for CT26 cells treated with LNT, UA or LNT-UA for 24 h. Scale bar = 100 µm. **(D)** Representative flow cytometric analysis of Annexin V-FITC/PI staining for CT26 cells treated with LNT, UA or LNT-UA for 24 h.

To evaluate the anti-cancer effect of LNT-UA *in vitro*, we first detected the cell viability of murine colorectal cancer cell line CT26 cells at different concentrations by CCK-8 assay. After 24 h of treatment, we found that the viability of free UA and LNT-UA gradually decreased with the increase of UA concentration (Figure 2A). LNT-UA showed an obvious inhibitory effect on cell viability when UA concentration was greater than 16 μmol/L. In contrast, the cytotoxicity of free UA was much lower than LN-UA at the same dose of UA, which might due to the enhanced cellular uptake after assembly into nanoparticles. Whereas, LNT alone did not show any significant cytotoxicity. Finally, 22 μmol/L of UA was selected as the concentration for subsequent experiments. To identify whether the higher cytotoxicity of LNT-UA than free UA at the same concentration is resulted from the increased drug bioavailability, we detected UA uptake by CT26 cells after incubation with free UA and LNT-UA for 4 h and 8 h by HPLC. As expected, Figure 2B showed that the much higher UA in the LNT-UA treated group was taken into CT26 cells in comparison with free UA after treatment for 8 h at the same concentration.

In order to display the cytotoxicity of LNT-UA to CT26 cells more intuitively, the cells were stained with Live/Dead staining kit and observed by fluorescence microscope. Consistently, in Figure 2C, LNT-UA caused large number of dead cells with red fluorescence, which was much higher than other three groups. To further confirm whether the cell death induced by LNT-UA was related to apoptosis, Annexin V-FITC/PI assay was employed to analyze cell apoptosis after drug intervention for 24 h. As described in Figure 2D, LNT had a negligible effect on CT26 cells. Compared to the percentage of apoptosis in the UA treated group (32.22%), there was a much higher apoptosis rate (66.35%) in the LNT-UA treated group. These results indicated that the as-prepared LNT-UA could improve the bioavailability and promote cell killing effect associated with apoptosis pathway.

Moreover, we further explored the *in vitro* cytotoxicity of free UA to APC cells (BMDMs and BMDCs) by CCK-8 assay. As shown in Figure S5, UA also exhibited an inhibiting effect on APCs to some extent. However, in the scenario of *in vivo* antitumor effect, more APCs may be recruited into the initial tumor site, and the tumor-associated antigens in tumor debris post treatment may be transported into nearby lymph nodes and then processed by APCs to stimulate immune response, although APCs pre-existing in the tumor might have been killed alongside the tumor 33, 34.

### LNT-UA induces ICD

**Figure 3:**
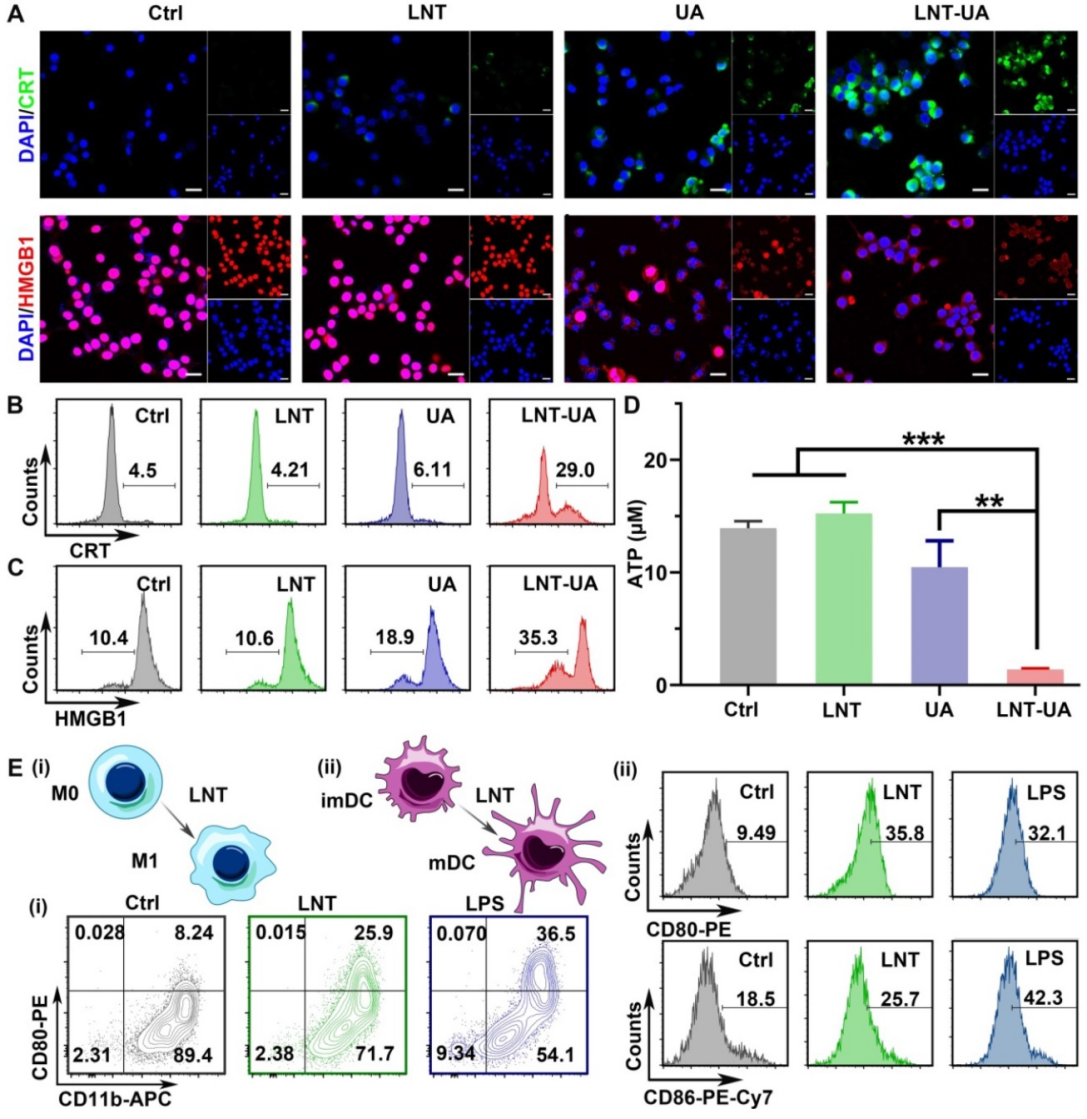
** LNT-UA induced ICD. (A)** Representative immunofluorescence images of CRT (green) or HMGB1 (red) expression of CT26 cells with different treatments, cell nucleus was stained with DAPI. Scale bar = 20 µm. **(B, C)** Flow cytometry of CRT (B) or HMGB1 (C) expression of CT26 cells. **(D)** Intracellular ATP content measurement of CT26 cells treated with different drugs. Data are presented as mean ± S.D (n = 3). **P < 0.01, ***P < 0.001. **(E)** Representative flow cytometric analysis of macrophage polarization (i) and BMDC maturation (ii) with the treatment of LNT for 48 h. LPS was used as the positive control.

Besides the release of TAAs, tumor cells with immunogenic cell death have other distinct characteristics: CRT exposure on the cell membrane as a “eat me” signal to facilitate phagocytosis, HMGB1 release from nucleus to effectively promote the maturation of APCs, and ATP release as a “find me” signal to recruit more immune cells 35, 36. Thus, to evaluate whether LNT-UA can induce ICD of CT26 cells, the expression of CRT and HMGB1 were firstly detected by immunofluorescence staining. According to the results in Figure 3A, the cells in both the control and LNT treated groups had weak fluorescence of CRT in the cytoplasm and intense fluorescence of HMGB1 in nucleus, respectively. In contrast, CRT exposure became more evident on the cell membrane while HMGB1 signal in nucleus turned to decline in the UA treated group. Notably, after treatment of LNT-UA, CRT exposure was more distinctly observed on the cell membrane surface of CT26 cells. Meanwhile, the expression of HMGB1 in the nucleus was also sharply reduced. Similar results were also obtained by flow cytometry (Figure 3B-C). Meanwhile, the cells treated with LNT-UA were demonstrated to significantly release ATP into the extracellular environment (Figure 3D). Collectively, these results proved that LNT-UA could induce ICD of CT26 cells efficiently, which could elicit downstream immune responses.

The immune response triggered by drug-induced immunogenic cell death alone is usually weak, while the combination with an immunomodulator can amplify the antitumor immunity. LNT has been widely used in studies as an immune adjuvant, so we checked the effect of LNT on mouse BMDMs and mouse BMDCs by flow cytometry (Figure 3E and Figure S6-7). Compared with the control group, the macrophages after LNT treatment showed a higher expression of CD80 (M1 marker) like the LPS positive group, suggesting that LNT can polarize M0 macrophages to M1 (CD11b^+^CD80^+^ cells). However, the polarization effect of LNT-UA was less pronounced than LNT (Figure 3E and Figure S8), which might be due to the decreased activity of LNT to recognize PRRs 23, as well as the increased cytotoxicity against APCs after assembled with UA (Figure S5). To further explore the macrophage polarization effect of LNT, the BMDM cells were firstly treated with interleukin 4 (IL-4) to induce M2 phenotype 37, and then incubate with LNT. As shown in Figure S9, the ratio of M1 phenotype (CD11b^+^CD80^+^) was increased significantly from 8.8% to 31.0%, while M2 phenotype (CD11b^+^CD206^+^) decreased from 68.7% to 43.0% after incubation with LNT. Additionally, LNT also significantly promoted the expression of CD80 (35.8%) and CD86 (25.7%) on the BMDC surface, indicating its role to stimulate the maturation of BMDCs. Taken together, LNT not only can serve as a carrier to increase the bioavailability of UA but also has the ability to stimulate the maturation of DCs and induce BMDMs to polarize toward M1, which benefits to enhance the ICD immune response.

### *In vivo* antitumor effect

**Figure 4:**
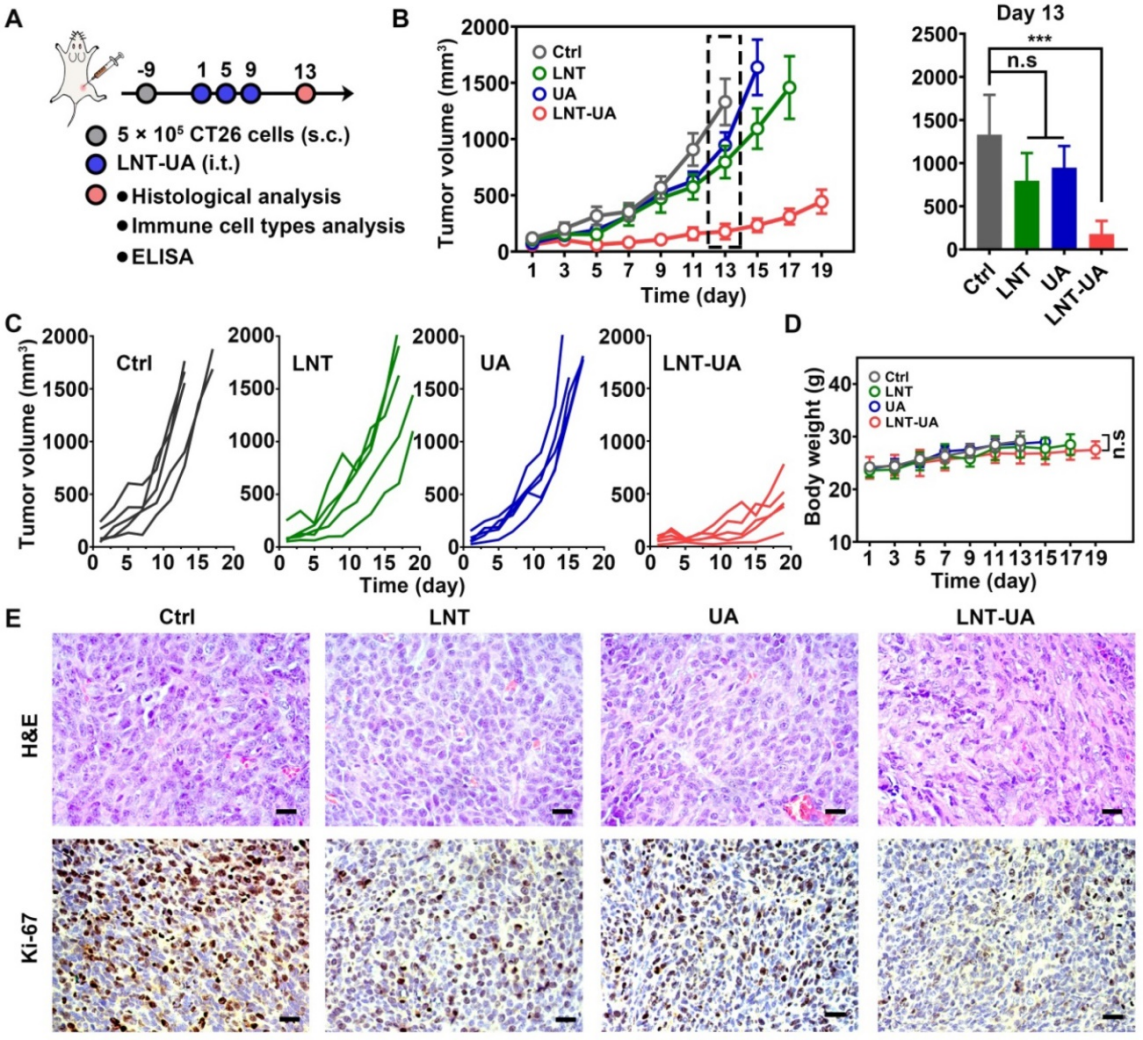
** LNT-UA inhibited tumor growth in CT26 tumor model. (A)** Schematic diagram of experimental plan. **(B, C)** The tumor growth curves of saline, LNT, UA or LNT-UA. Data are presented as mean ± S.E.M (n = 5). ***P < 0.001. **(D)** The body weight of mice during experiment. Data are presented as mean ± S.D (n = 5). **(E)** Representative H&E staining and immunochemistry images of Ki-67 of tumor tissue from mice. Scale bar = 20 µm. *P < 0.05, ***P < 0.001.

Encouraged by the remarkable tumor suppressive effects of LNT-UA *in vitro*, we next sought to investigate the antitumor effect of LNT-UA on subcutaneous transplanted CT26 cells in BALB/c mice. Four days after the 3^rd^ administration, a portion of mice was euthanatized for detecting the relative indexes, as illustrated in Figure 4A. The tumor volume of tumor-bearing mice was monitored every other day. As depicted in Figure 4B-C, LNT and free UA had moderate tumor suppression effect, in comparison with the saline group, which might be ascribed to the immune stimulation and ICD effect, respectively. In contrast, the tumor growth was significantly slowed down after the administration of LNT-UA, due to the enhanced UA bioavailability and combination effect of LNT and UA. During the course of treatment, LNT-UA had no significant influence on the body weight of mice, suggesting limited side effects of this therapeutic paradigm (Figure 4D). The H&E staining of tumor tissues suggested that LNT-UA caused intensive tumor cell necrosis with nuclear shrinkage or even disappearance, while LNT and UA showed no significant changes as compared with the control group (Figure 4E). Ki-67, as a nuclear antigen reflecting the proliferation activity of tumor cells, was also checked. Compared with other three groups, the expression of Ki-67 was signally lower in tumor sections of LNT-UA treated group. To further verify the superiority of co-delivery of UA and LNT by LNT-UA, the *in vitro* and *in vivo* anticancer effect of the physical mixture of LNT and UA (LNT+UA) has been investigated and compared with LNT-UA, as shown in Figure S10. The results demonstrated that LNT-UA exhibited a more pronounced anticancer effect than LNT+UA, probably due to the enhanced cellular uptake after assembly into nanoparticles (Figure 2B). All of the above results indicate the great potential of nanodrug resulting from the traditional Chinese medicine of LNT and UA for anti-CRC therapy.

**Figure 5:**
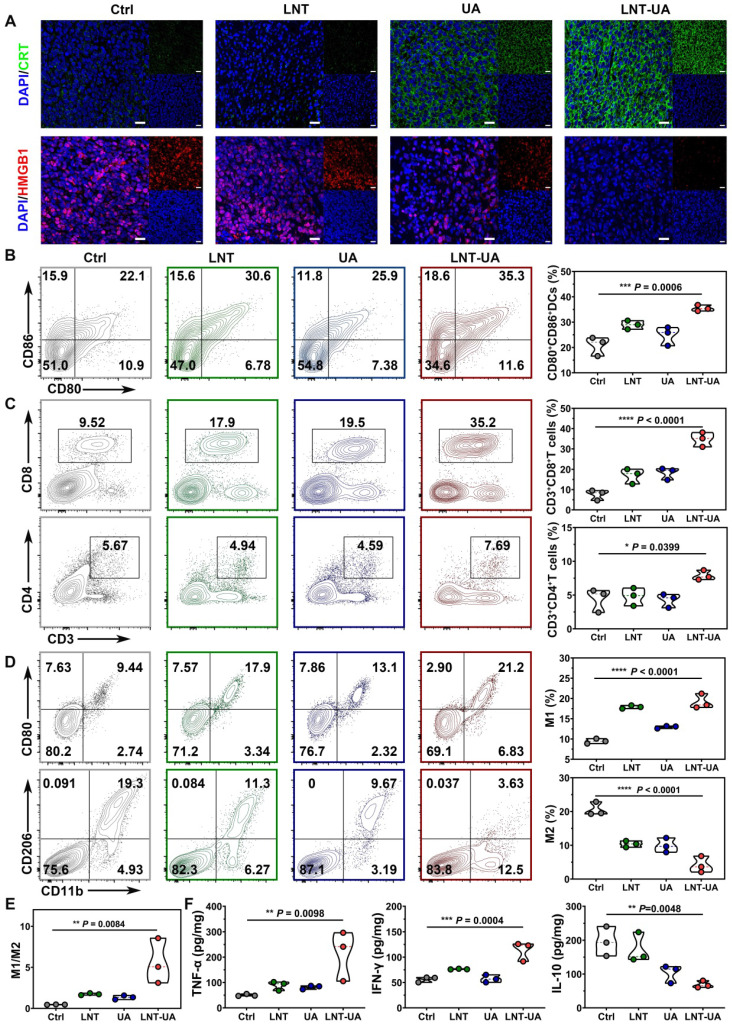
** LNT-UA induced immune effects by regulating tumor microenvironment. (A)** Representative immunofluorescence images of CRT (green) and HMGB1 (red) of tumor tissue from mice. Scale bar = 20 µm. **(B)** Percentages of CD80^+^CD86^+^ DC cells in TDLNs by flow cytometry (n = 3). **(C)** Percentages of tumor infiltrating effector T cells by flow cytometry (n = 3). **(D, E)** Percentages of tumor infiltrating M1/M2 macrophages by flow cytometry (n = 3).** (F)** Intra-tumoral cytokine levels of TNF-α, IFN-γ and IL-10 by ELISA (n = 3). Data are presented as mean ± S.D. *P < 0.05, **P < 0.01, ***P < 0.001, ****P < 0.0001.

To explore the underlying mechanism of the antitumor effect of LNT-UA *in vivo*, CRT and HMGB1 expression were analyzed by immunofluorescent staining of tumor slides. As observed in Figure 5A, after treatment with LNT-UA, the expression of CRT on tumor cell surface increased significantly, while HMGB1 in the nucleus decreased significantly, indicating that the LNT-UA also can induce immunogenic death of tumor cells *in vivo*. Immature DCs would uptake TAAs released from dying tumor cells, and became matured in the TDLNs to present antigen to naïve T cells. Therefore, we analyzed the maturation of DCs in TDLNs by flow cytometry. The results in Figure 5B and Figure S11 exhibited that the percentage of matured DCs in the mice receiving LNT-UA treatment had a remarkable increase to 35.3%, which was more efficient than other groups.

Next, to verify whether LNT-UA can exert antitumor immunity in tumor tissue through reverse the immunosuppression of TME, we firstly analyzed the infiltration of T cells and macrophages in tumor tissue by flow cytometry. T cells are the main immune cells that play the role of antitumor immunity but are often excluded out by suppressive TME. Meanwhile, tumor-associated macrophages (TAMs) accounting for the largest number of immune cells in tumors tend to be polarized into a tumorigenic M2-like phenotype 38. The results in Figure 5C and Figure S12-13 showed that LNT-UA treatment improved the intra-tumoral infiltration of CD3^+^CD8^+^ T cells and CD3^+^CD4^+^ T cells, which were nearly 2-fold and 4-fold higher in comparison with the control group, respectively. Figure 5D and Figure S14-15 showed that the percentages of M1 (CD11b^+^CD80^+^ cells) in the tumor tissues of the LNT-UA group were markedly increased, while the percentages of M2 (CD11b^+^CD206^+^ cells) were decreased, leading to the upregulated ratio of M1/M2 macrophages (Figure 5E). Furthermore, LNT-UA induced immune response *in vivo* was confirmed by measuring cytokine secretion in tumor tissues. As shown in Figure 5F, the secretion of antitumor related cytokines (IFN-γ, TNF-α) was found to elevate, while the pro-tumorigenic cytokines of IL-10 declined after LNT-UA treatment. These data indicate that the antitumor effect of LNT-UA is closely related to the increased tumor cell immunogenicity by regulating the tumor microenvironment to stimulate innate and adaptive immune responses.

### LNT-UA together with αCD47 for CRC metastasis inhibition

**Figure 6:**
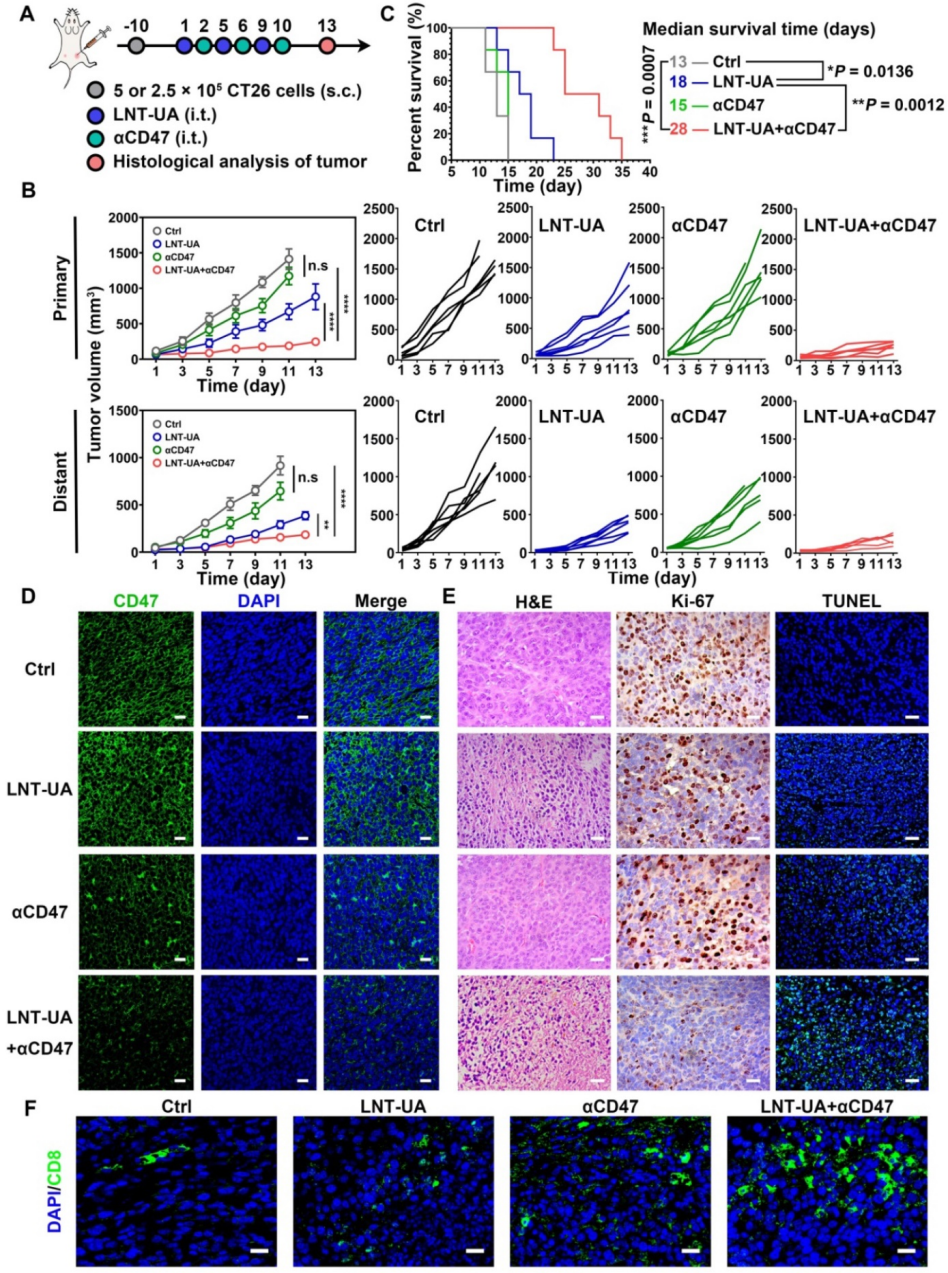
** LNT-UA together with αCD47 achieved abscopal effect in bilateral tumor model. (A)** Schematic diagram of experimental plan. **(B)** The primary and distant tumor growth curves of saline, LNT-UA, αCD47 and LNT-UA + αCD47. Data are presented as mean ± S.E.M (n = 6). **P < 0.01, ****P < 0.0001. **(C)** Survival curves of bilateral tumor-bearing mice (n = 6). *P < 0.05, **P < 0.01, ***P < 0.001. **(D)** Representative immunofluorescence images of CD47 (green) in primary tumor tissue. Scale bar = 20 µm. **(E)** H&E staining (scale bar = 20 µm), immunochemistry images of Ki-67 (scale bar = 20 µm) and TUNEL assay (scale bar = 40 µm) of distant tumor tissue from mice. **(F)** Representative immunofluorescence images of CD8 (green) in distant tumor tissue. Scale bar = 20 µm.

Although LNT-UA obviously delayed tumor growth, most of the mice (4 out of 5) still experienced further growth after treatment (Figure 4C), which might be due to the overactivation of other negative immune regulators in the TME to develop resistance to or even evade from immune surveillance. For instance, tumor cells can upregulate the “don't eat me” signal of CD47 to protect them from CRT-mediated phagocytosis elicited by ICD, which was also proved in our data 39. Thus, we sought to improve antitumor immune response by combining LNT-UA with αCD47. Meanwhile, to investigate whether their combinational antitumor effect could achieve an “*in situ* vaccination response” to suppress distant micro-metastases, we established a bilateral subcutaneous graft tumor model in BALB/c mice with primary tumor treated by various formulations as a tumor vaccine. LNT-UA was injected intratumorally at day 1, 5, 9, while αCD47 was also intratumorally injected into the primary tumor to prevent non-specific binding with platelets and red blood cells in the blood at day 2, 6, 10 (Figure 6A). It could be seen in Figure 6B that LNT-UA + αCD47 showed the most dramatic suppression of both primary and distant tumor growth, as well as significantly prolonged the survival time of mice than other groups (Figure 6C), indicating the treated primary tumor elicited a strong vaccination and systemic immune response against distant/spread tumor cells. After treatments, the body weight of mice had no significant changes among groups (Figure S16). To elucidate the corresponding molecular mechanism, CD47 expression after treatments was checked by immunofluorescence staining. As illustrated in Figure 6D, CD47 was found to obviously upregulate in tumor tissues of LNT-UA-treated mice but was effectively blocked by αCD47 in LNT-UA + αCD47 treated group.

We then evaluated the antitumor immune effect of LNT-UA + αCD47 on the distant tumor by histological methods. Compare with other three groups, the LNT-UA + αCD47 group showed obviously increased tumor cell necrosis, reduced Ki-67 expression, as well as elevated positive TUNEL rate (Figure 6E). The results of immunofluorescence of CD8 showed more infiltration of CD8^+^ T cells in the combined group (Figure 6F). Collectively, the combination of LNT-UA and αCD47 significantly restrained the primary tumor and distant tumor growth and extended the overall survival of mice, achieving the abscopal effect by cytotoxic T lymphocyte (CTL)-mediated antitumor immune response.

### Treatment with LNT-UA together with αCD47 for spontaneous CRC model

**Figure 7:**
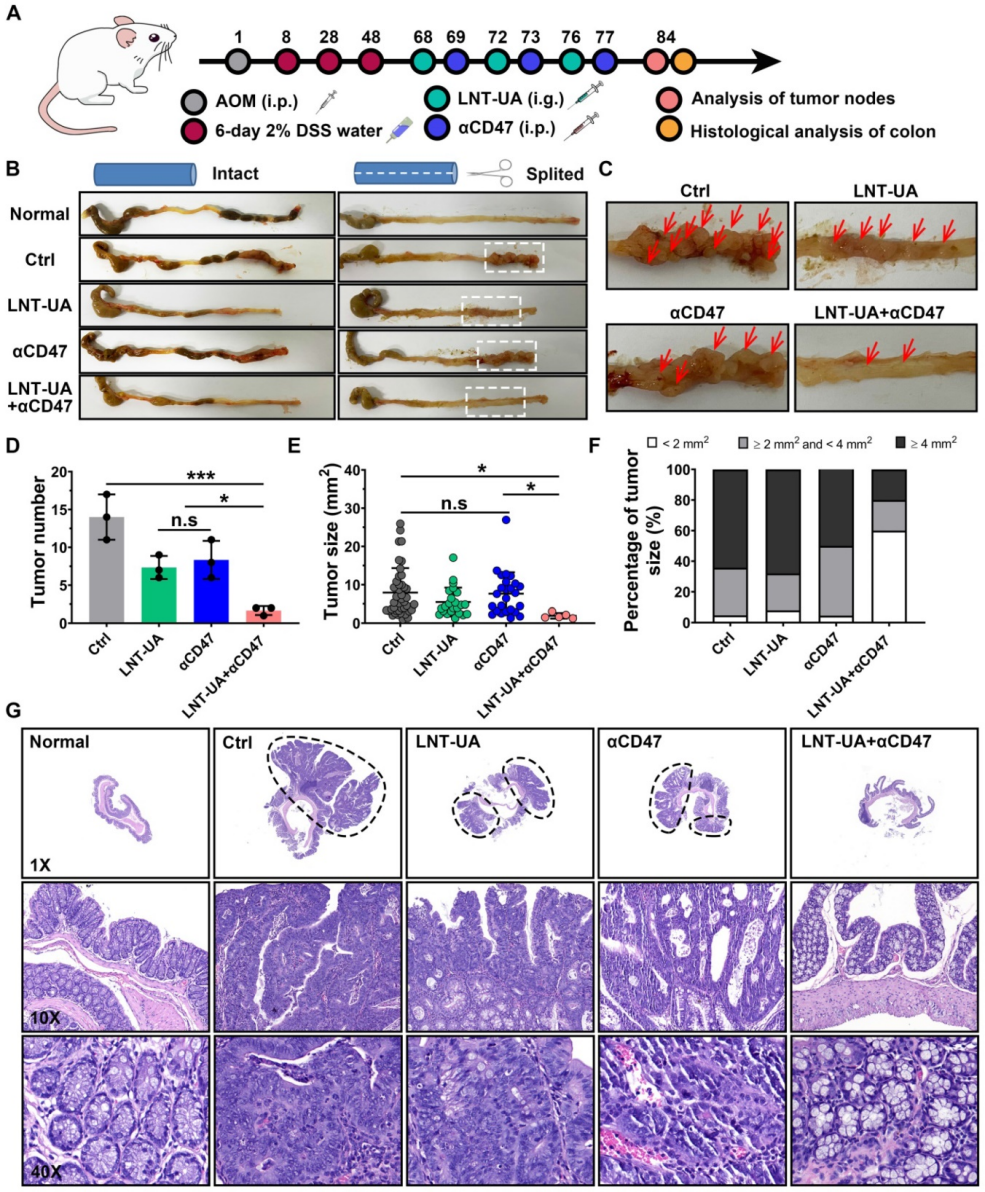
** LNT-UA together with αCD47 inhibited tumor growth in spontaneous CRC model. (A)** Schematic diagram of experimental plan. **(B, C)** Representative colon pictures of different groups: (1) Normal, (2) Ctrl (Saline), (3) LNT+UA, (4) αCD47, (5) LNT-UA+αCD47. **(D-F)** The tumor number (D), sum of tumor size (E), and the percentage of different tumor sizes (F) of different groups. Data are presented as mean ± S.D. (n = 3). *P < 0.05, ***P < 0.001. (G) H&E staining of colonic epithelium of different groups.

To better mimic the microenvironment in which CRC cells grow in the intestinal tract, a spontaneous CRC model was established by administration with AOM + DSS to evaluate the therapeutic efficacy of LNT-UA plus αCD47 40. Spontaneous CRC murine models were treated with LNT-UA by oral gavage at day 68, 72 and 76 (Figure 7A). The αCD47 were administered to the mice by i.p. injection at day 69, 73 and 77. Because the LNT-UA nanodrug is administrated via oral gavage, we firstly verified the stability of the LNT-UA nanodrug in the highly acidic environment of the stomach. As shown in Figure S17, there was still a substantial portion of LNT-UA maintained nanostructure after storing at pH 2 (to mimic a highly acidic environment of the stomach) for 2 h (gastric emptying time), implying that some nanoparticles can go through the stomach and arrive in the intestine and then internalized by cancer cells. Tumors in colons collected from mice on day 84 were photographed, counted and measured for determining the therapeutic effect. As shown in Figure 7B-C, tumor burden significantly decreased after the treatment of LNT-UA + αCD47, compared to other groups. The numbers of tumor nodules were calculated to be 1.67 ± 0.58, 7.33 ± 1.52, 7.67 ± 3.06, and 14.00 ± 3.00, in the group of LNT-UA + αCD47, single LNT-UA, single αCD47, and the control group, respectively (Figure 7D). In addition, the tumor size was also found to dramatically decline after treatment with LNT-UA + αCD47 (Figure 7D), with much less average tumor area compared with other groups. Moreover, we specifically classified these tumor nodules into three sections according to their size, that is, larger tumor (≥ 4 mm^2^), middle tumor (≥ 2 mm^2^ and <4 mm^2^), and smaller tumor (<2 mm^2^) 41. More remarkably, the results in Figure 7E verified that the LNT-UA+αCD47 could obviously delay the tumor progression from smaller to more aggressive larger ones. Meanwhile, histopathological analysis (H&E) of colonic epithelium further revealed that the structure of colonic mucosa glands of mice in the LNT-UA + αCD47 group recovered to be a similar regular state as the normal group, whereas the H&E images of colons from other groups showed a large tumor area with distinct adenocarcinomas feature (Figure 7G). Collectively, these findings clearly proved that LNT-UA plus αCD47, arising from the theory in traditional Chinese medicine, could effectively reshape the immunosuppressive tumor microenvironment to a more immunogenic one with plenty of effector T cells and M1-TAM to prevent CRC formation and aggression.

**Figure 8:**
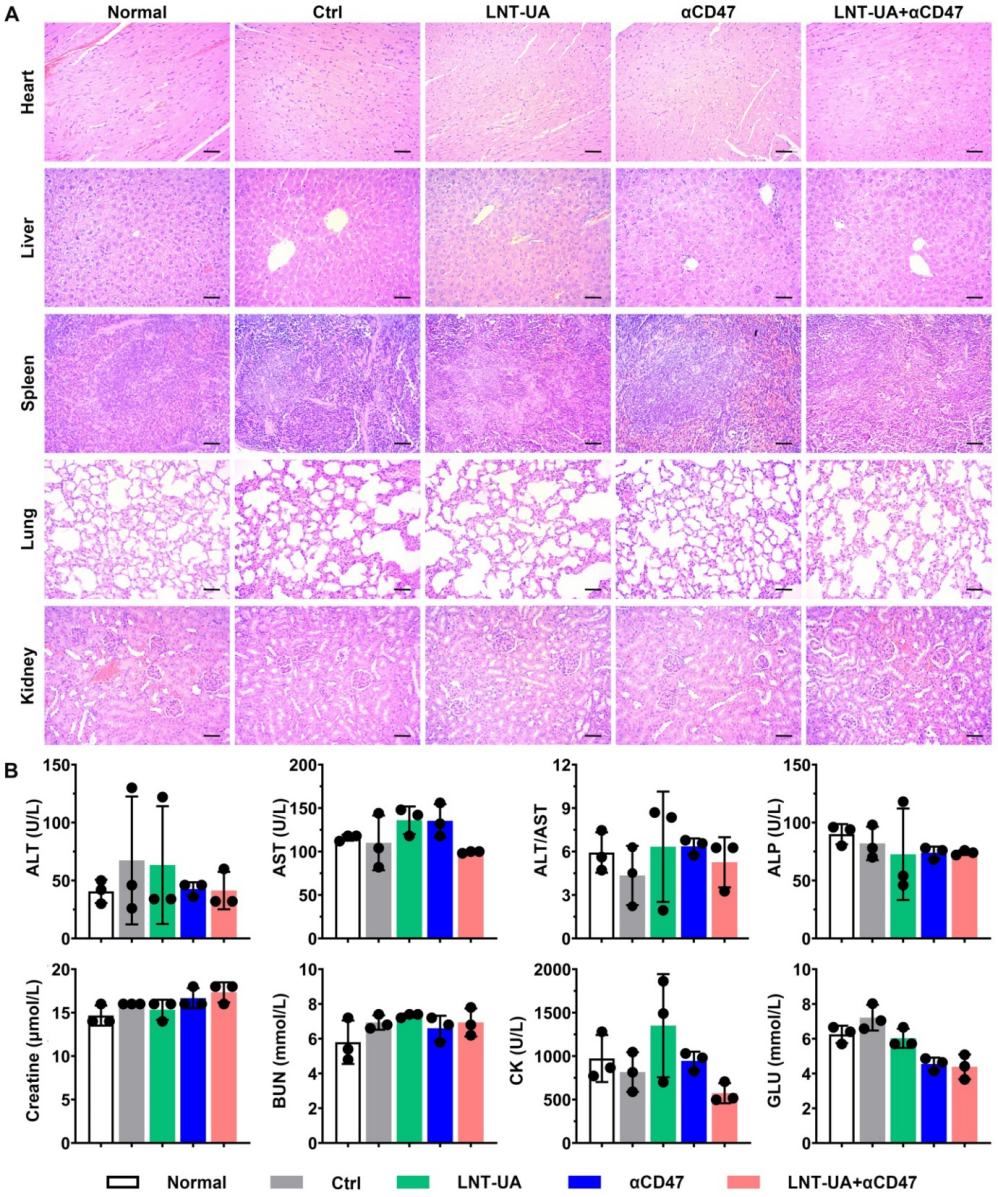
** Biosafety assay of different treatment *in vivo*. (A)** H&E staining of major organs. Scale bar = 50 µm. **(B)** Serum biochemical indicator test. Data are presented as mean ± S.D. (n = 3).

To further evaluate the biosafety of LNT-UA plus αCD47 *in vivo*, major organs and serum were collected for histological and biochemical indicator detection at the endpoint. The results of H&E staining demonstrated no significant toxic damage in major organs of the mice in the LNT-UA + αCD47 group (Figure 8A). The relative index of liver and kidney function, including ALT, AST, ALP, Creatine, and BUN, was not changed after LNT-UA+αCD47 administration. Meanwhile, myocardial enzyme and glucose in mice serum were also found to maintain normal levels (Figure 8B), indicating the treatment of LNT-UA+αCD47 had no obvious systemic toxicity in mice.

## Conclusions

In summary, we herein presented a natural product nano-formulation (LNT-UA) by the self-assembly of LNT and UA based on the theory of “strengthening the body (*Fu Zheng*) or eliminating evil (*Qu Xie*)” in traditional Chinese medicine with a long history, to mediate immunotherapy for CRC. The nanodrug displayed spherical morphology with narrow size distribution and superior stability in aqueous solution, with an extremely high drug-loading rate as no extra carriers were used. Compared with free UA, LNT-UA displayed an improved drug bioavailability and anticancer performance, which then induced ICD response to increase tumor immunogenicity. In addition to being a solubilizer for UA, LNT in the nanodrug was demonstrated to further act as an immunologic adjuvant to promote ICD response, thereby activating DCs, polarizing TAMs towards M1, recruiting effector T cells, and increasing antitumor related cytokine levels. Following the principle of an integrative theory in TCM on overall regulation, the further combination of LNT-UA and αCD47 was demonstrated to more efficiently reinforce the antitumor immunity to effectively inhibit tumor growth and metastasis, as proved in multiple tumor models including bilateral and spontaneous CRC tumors. To our knowledge, this is the first report on the use of the co-assembly of LNT and UA for CRC treatment, and our findings may stimulate the development of various TCM-based formulations based on TCM theory. Thus, this relatively facile, scalable, and modernized natural product formulation (*Fufang*, LNT-UA) based on TCM theory focusing on the overall regulation offers a promising strategy for CRC immunotherapy.

## Supplementary Material

Supplementary figures and table.

## Abbreviations

CRC: colorectal cancer
ICB: checkpoint blockade
ICD: immunogenic cell death
TAAs: tumor-associated antigens
DAMPs: damage-associated molecular patterns
TCM: traditional Chinese medicines
UA: ursolic acid
ER: endoplasmic reticulum
CFND: carrier-free nanodrug
LNT: Lentinan
PRRs: pattern recognition receptors
TLRs: toll-like receptors
APCs: antigen-presenting cells
NF-κB: nuclear factor kappa-B
TME: tumor microenvironment
SIRPα: signal regulatory protein alpha
αCD47: anti-CD47 antibody
BCA: bicinchoninic acid
CCK-8: cell counting kit-8
ATP: adenosine triphosphate
AOM: azoxymethane
DSS: dextran sulfate sodium
GM-CSF: granulocyte macrophage colony-stimulating factors
IL-4: interleukin-4
ELISA: enzyme-linked immunosorbent assay
DMSO: dimethyl sulfoxide
MWCO: molecular weight cut-off
TEM: transmission electron microscope
SEM: scanning electron microscopy
FT-IR: fourier transform infrared
DLS: dynamic light scattering
HPLC: high-performance liquid chromatography
RIPA: radio immunoprecipitation assay
CRT: calreticulin
HMGB1: high mobility group protein 1
CLSM: confocal laser scanning microscope
BMDCs: bone marrow dendritic cells
BMDMs: bone marrow-derived macrophages
H&E: hematoxylin-eosin
TDLNs: tumor-draining lymph nodes
IL-10: interleukin-10
IFN-γ: interferon-γ
TNF-α: tumor necrosis factor-α
ANOVA: one-way analysis of variance
DC: Dendritic cell
CTL: cytotoxic T lymphocyte

## References

[1] Sung H, Ferlay J, Siegel RL, Laversanne M, Soerjomataram I, Jemal A (2021). Global cancer statistics 2020: GLOBOCAN estimates of incidence and mortality worldwide for 36 cancers in 185 countries. CA Cancer J Clin.

[2] Bagchi S, Yuan R, Engleman EG (2021). Immune checkpoint inhibitors for the treatment of cancer: clinical impact and mechanisms of response and resistance. Annu Rev Pathol.

[3] Ganesh K, Stadler ZK, Cercek A, Mendelsohn RB, Shia J, Segal NH (2019). Immunotherapy in colorectal cancer: rationale, challenges and potential. Nat Rev Gastroenterol Hepatol.

[4] Gutting T, Burgermeister E, Hartel N, Ebert MP (2019). Checkpoints and beyond - immunotherapy in colorectal cancer. Semin Cancer Biol.

[5] Krysko DV, Garg AD, Kaczmarek A, Krysko O, Agostinis P, Vandenabeele P (2012). Immunogenic cell death and DAMPs in cancer therapy. Nat Rev Cancer.

[6] Duan X, Chan C, Lin W (2019). Nanoparticle-mediated immunogenic cell death enables and potentiates cancer immunotherapy. Angew Chem Int Ed Engl.

[7] Huehnchen P, van Kampen A, Boehmerle W, Endres M (2020). Cognitive impairment after cytotoxic chemotherapy. Neuro-oncol Pract.

[8] Deng L, Qi M, Li N, Lei Y, Zhang D, Chen J (2020). Natural products and their derivatives: promising modulators of tumor immunotherapy. J Leukoc Biol.

[9] Zhang J, Shen L, Li X, Song W, Liu Y, Huang L (2019). Nanoformulated codelivery of quercetin and alantolactone promotes an antitumor response through synergistic immunogenic cell death for microsatellite-stable colorectal cancer. ACS Nano.

[10] Yu Z, Guo J, Hu M, Gao Y, Huang L (2020). Icaritin exacerbates mitophagy and synergizes with doxorubicin to induce immunogenic cell death in hepatocellular carcinoma. ACS Nano.

[11] Zhang N, Liu S, Shi S, Chen Y, Xu F, Wei X (2020). Solubilization and delivery of ursolic-acid for modulating tumor microenvironment and regulatory T cell activities in cancer immunotherapy. J Control Release.

[12] Zheng Q, Li P, Jin F, Yao C, Zhang G, Zang T (2013). Ursolic acid induces ER stress response to activate ASK1-JNK signaling and induce apoptosis in human bladder cancer T24 cells. Cell Signal.

[13] Gou W, Luo N, Wei H, Wu H, Yu X, Duan Y (2020). Ursolic acid derivative UA232 evokes apoptosis of lung cancer cells induced by endoplasmic reticulum stress. Pharm Biol.

[14] Kim K, Shin EA, Jung JH, Park JE, Kim DS, Shim BS (2018). Ursolic acid induces apoptosis in colorectal cancer cells partially via upregulation of microRNA-4500 and inhibition of JAK2/STAT3 phosphorylation. Int J Mol Sci.

[15] Khwaza V, Oyedeji OO, Aderibigbe BA (2020). Ursolic acid-based derivatives as potential anti-cancer agents: an update. Int J Mol Sci.

[16] Deng S, Shanmugam MK, Kumar AP, Yap CT, Sethi G, Bishayee A (2019). Targeting autophagy using natural compounds for cancer prevention and therapy. Cancer.

[17] Zhang B, Jiang J, Wu P, Zou J, Le J, Lin J (2021). A smart dual-drug nanosystem based on co-assembly of plant and food-derived natural products for synergistic HCC immunotherapy. Acta Pharm Sin B.

[18] Wang M, Zhao T, Liu Y, Wang Q, Xing S, Li L (2017). Ursolic acid liposomes with chitosan modification: promising antitumor drug delivery and efficacy. Mater Sci Eng C Mater Biol Appl.

[19] Jiang K, Chi T, Li T, Zheng G, Fan L, Liu Y (2017). A smart pH-responsive nano-carrier as a drug delivery system for the targeted delivery of ursolic acid: suppresses cancer growth and metastasis by modulating P53/MMP-9/PTEN/CD44 mediated multiple signaling pathways. Nanoscale.

[20] Zhao R, Zheng G, Fan L, Shen Z, Jiang K, Guo Y (2018). Carrier-free nanodrug by co-assembly of chemotherapeutic agent and photosensitizer for cancer imaging and chemo-photo combination therapy. Acta Biomater.

[21] Xu Y, Mu J, Xu Z, Zhong H, Chen Z, Ni Q (2020). Modular acid-activatable acetone-based ketal-linked nanomedicine by dexamethasone prodrugs for enhanced anti-rheumatoid arthritis with low side effects. Nano Lett.

[22] Yu N, Liu T, Zhang X, Gong N, Ji T, Chen J (2020). Dually enzyme- and acid-triggered self-immolative ketal glycoside nanoparticles for effective cancer prodrug monotherapy. Nano Lett.

[23] Meng Y, Lyu F, Xu X, Zhang L (2020). Recent advances in chain conformation and bioactivities of triple-helix polysaccharides. Biomacromolecules.

[24] Chihara G, Maeda Y, Hamuro J, Sasaki T, Fukuoka F (1969). Inhibition of mouse sarcoma 180 by polysaccharides from Lentinus edodes (Berk.) sing. Nature.

[25] Deng S, Zhang G, Kuai J, Fan P, Wang X, Zhou P (2018). Lentinan inhibits tumor angiogenesis via interferon gamma and in a T cell independent manner. J Exp Clin Cancer Res.

[26] Zhang Y, Zhang M, Jiang Y, Li X, He Y, Zeng P (2018). Lentinan as an immunotherapeutic for treating lung cancer: a review of 12 years clinical studies in China. J Cancer Res Clin Oncol.

[27] Palao-Suay R, Gómez-Mascaraque LG, Aguilar MR, Vázquez-Lasa B, Román JS (2016). Self-assembling polymer systems for advanced treatment of cancer and inflammation. Prog Polym Sci.

[28] Lin M, Yang Z, Yang Y, Peng Y, Li J, Du Y (2022). CRISPR-based *in situ* engineering tumor cells to reprogram macrophages for effective cancer immunotherapy. Nano Today.

[29] Yan Z, Wang Q, Liu X, Peng J, Li Q, Wu M (2018). Cationic nanomicelles derived from Pluronic F127 as delivery vehicles of Chinese herbal medicine active components of ursolic acid for colorectal cancer treatment. RSC Adv.

[30] Wu M, Zheng D, Zhang D, Yu P, Peng L, Chen F (2020). Converting immune cold into hot by biosynthetic functional vesicles to boost systematic antitumor immunity. iScience.

[31] Li Y, Lin J, Cai Z, Wang P, Luo Q, Yao C (2020). Tumor microenvironment-activated self-recognizing nanodrug through directly tailored assembly of small-molecules for targeted synergistic chemotherapy. J Control Release.

[32] Yang F, Huang J, Liu H, Lin W, Li X, Zhu X (2020). Lentinan-functionalized selenium nanosystems with high permeability infiltrate solid tumors by enhancing transcellular transport. Nanoscale.

[33] Chen Q, Xu L, Liang C, Wang C, Peng R, Liu Z (2016). Photothermal therapy with immune-adjuvant nanoparticles together with checkpoint blockade for effective cancer immunotherapy. Nat Commun.

[34] Cheng Y, Chen Q, Guo Z, Li M, Yang X, Wan G (2020). An intelligent biomimetic nanoplatform for holistic treatment of metastatic triple-negative breast cancer via photothermal ablation and immune remodeling. ACS Nano.

[35] Wang S, Zhang Y (2020). HMGB1 in inflammation and cancer. J Hematol Oncol.

[36] Li J, Wang S, Lin X, Cao Y, Cai Z, Wang J (2022). Red blood cell-mimic nanocatalyst triggering radical storm to augment cancer immunotherapy. Nano-Micro Lett.

[37] Chang M, Hou Z, Jin D, Zhou J, Wang M, Wang M, Shu M, Ding B, Li C, Lin J (2020). Colorectal tumor microenvironment-activated bio-decomposable and metabolizable Cu_2_O@CaCO_3_ nanocomposites for synergistic oncotherapy. Adv Mater.

[38] Rao L, Wu L, Liu Z, Tian R, Yu G, Zhou Z (2020). Hybrid cellular membrane nanovesicles amplify macrophage immune responses against cancer recurrence and metastasis. Nat Commun.

[39] Chao MP, Jaiswal S, Weissman-Tsukamoto R, Alizadeh AA, Gentles AJ, Volkmer J (2010). Calreticulin is the dominant pro-phagocytic signal on multiple human cancers and is counterbalanced by CD47. Sci Transl Med.

[40] Neufert C, Becker C, Neurath MF (2007). An inducible mouse model of colon carcinogenesis for the analysis of sporadic and inflammation-driven tumor progression. Nat Protoc.

[41] Hwang S, Lee CG, Jo M, Park CO, Gwon SY, Hwang S (2020). Enterotoxigenic Bacteroides fragilis infection exacerbates tumorigenesis in AOM/DSS mouse model. Int J Med Sci.

