# Supplementary material for: Self-assembled traditional Chinese nanomedicine modulating tumor immunosuppressive microenvironment for colorectal cancer immunotherapy
Source: Theranostics. 2022 Aug 15;12(14):6088–105. doi: 10.7150/thno.72509 (PMC9475452; doi:10.7150/thno.72509)
Supplement: Supplementary file 1 — Supplementary figures and table. [file thnov12p6088s1.pdf]

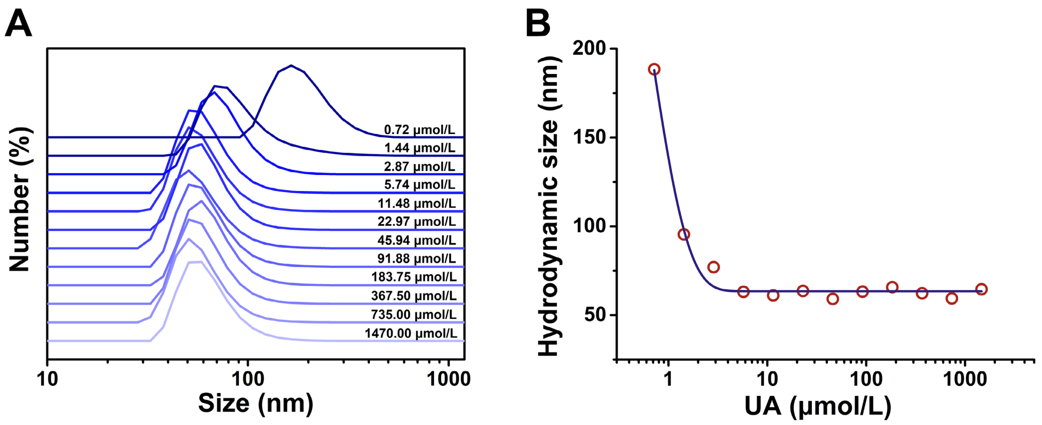

Figure S1. The hydrodynamic size change of LNT-UA nanodrug at different UA concentrations by DLS.

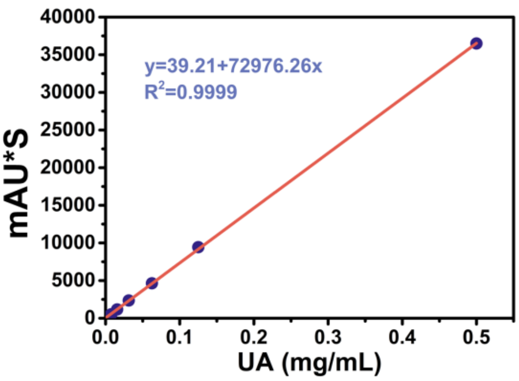

Figure S2. The standard curve of UA detected by HPLC. Related to Figure 1G-H.

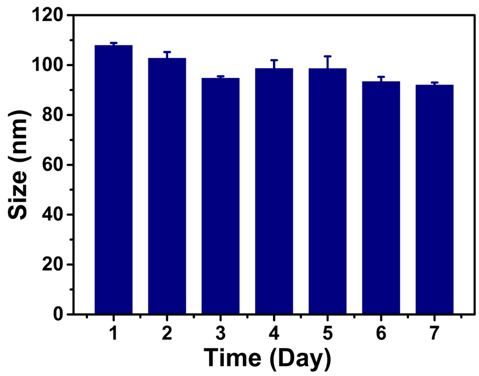

Figure S3. The hydrodynamic size of LNT-UA in phenol red-free DMEM supplemented with 10% (vol/vol) FBS for 7 days. Data are presented as mean ± S.D (n = 3).

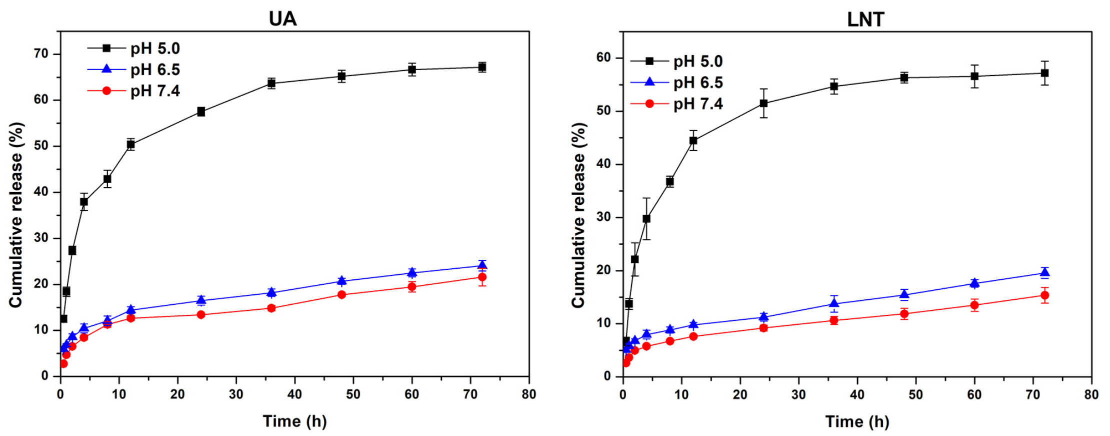

Figure S4. *In vitro* release behavior of UA and LNT from LNT-UA at different pH values (n = 3).

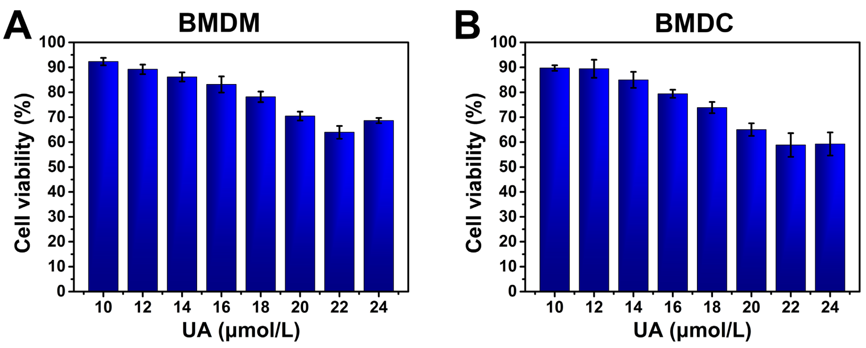

Figure S5. Cell viability of BMDM and BMDC cells treated with LNT-UA at different concentrations for 24 h.

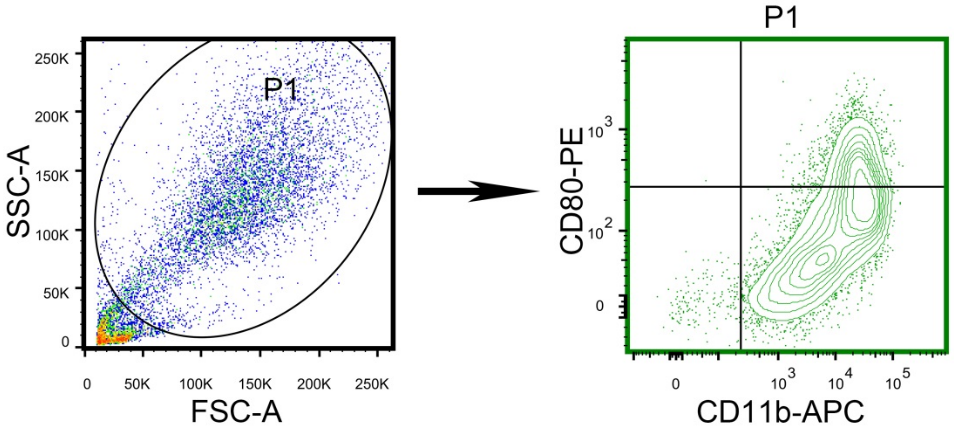

Figure S6. Representative flow cytometric gating strategy for detecting the percentage of M1 macrophages ($CD11b^+CD80^+$ cells). Related to Figure 3E.

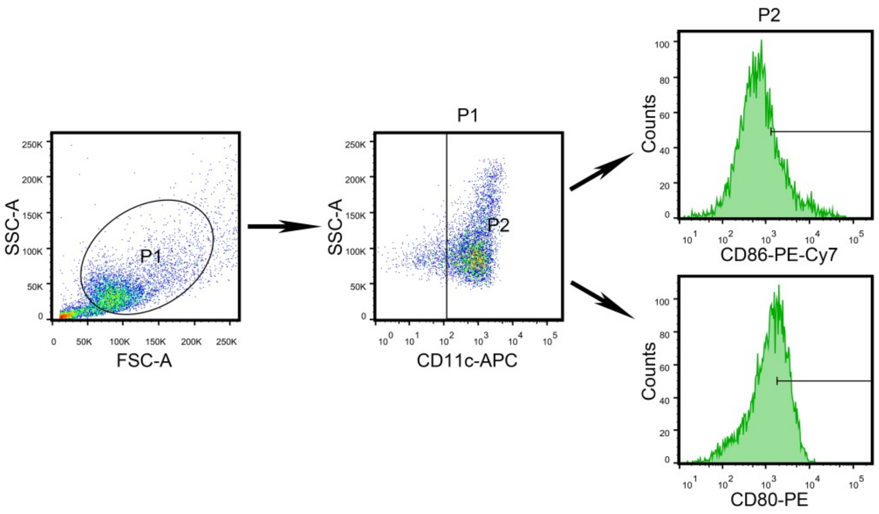

Figure S7. Representative flow cytometric gating strategy for detecting the percentage of matured dendritic cells ($CD11c^+CD80^+$ cells or $CD11c^+CD86^+$ cells). Related to Figure 3E.

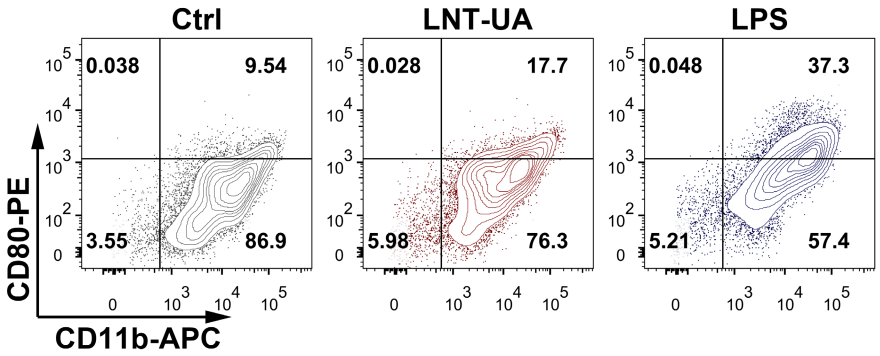

Figure S8. Representative flow cytometric analysis of macrophage polarization with the treatment of LNT-UA for 48 h. LPS was used as the positive control.

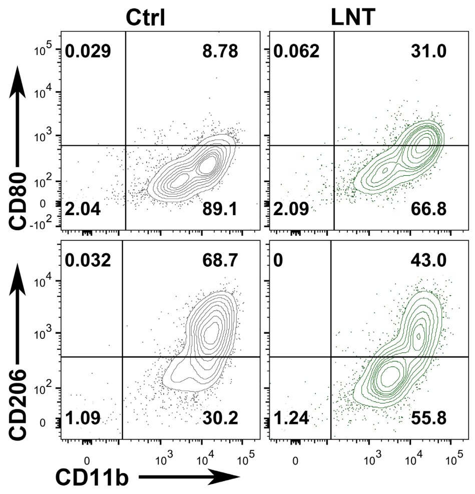

Figure S9. Representative flow cytometric analysis of the polarization of M2 macrophages after incubation with LNT.

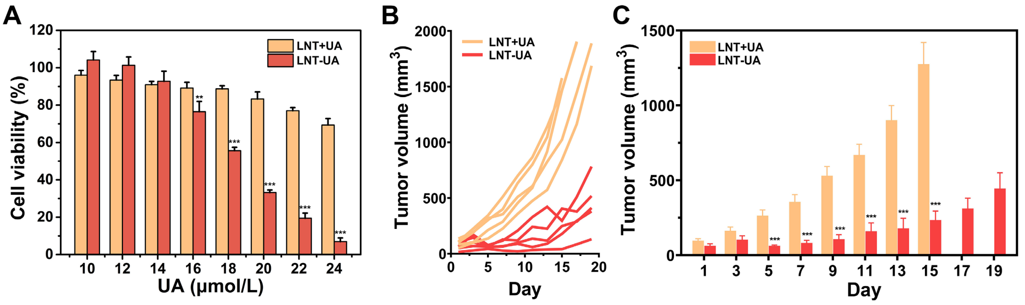

Figure S10. *In vitro* (A) and *in vivo* (B-C) anticancer effect of LNT-UA and LNT+UA. The data of the LNT-UA group is related to Figure 2A and Figure 4B-C.

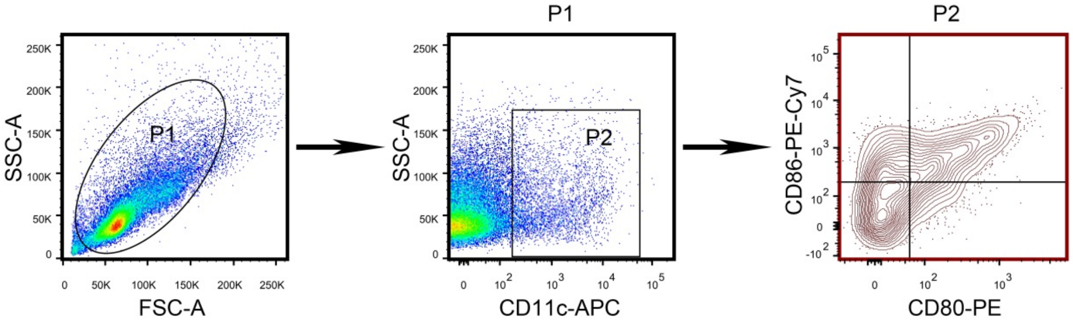

Figure S11. Representative flow cytometric gating strategy for detecting the percentage of CD80$^+$CD86$^+$ DCs in tumor draining lymph nodes (TDLNs). Related to Figure 5B.

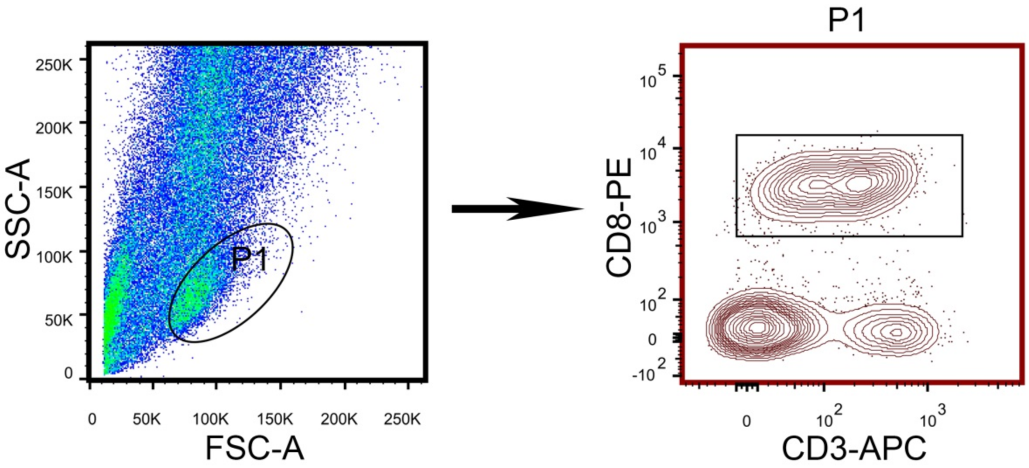

Figure S12. Representative flow cytometric gating strategy for detecting the percentage of tumor infiltrating CD8$^+$ T cells. Related to Figure 5C.

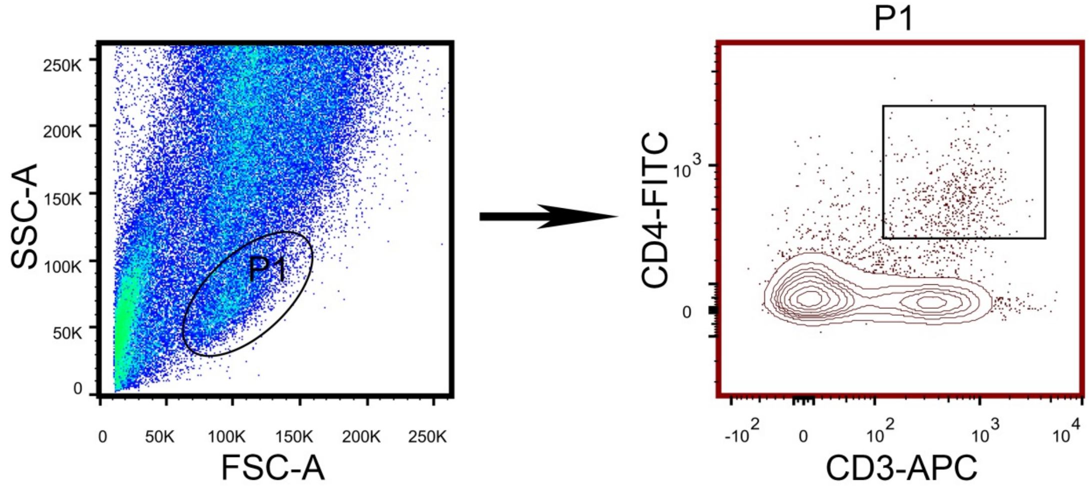

Figure S13. Representative flow cytometric gating strategy for detecting the percentage of tumor infiltrating CD4^{+} T cells. Related to Figure 5C.

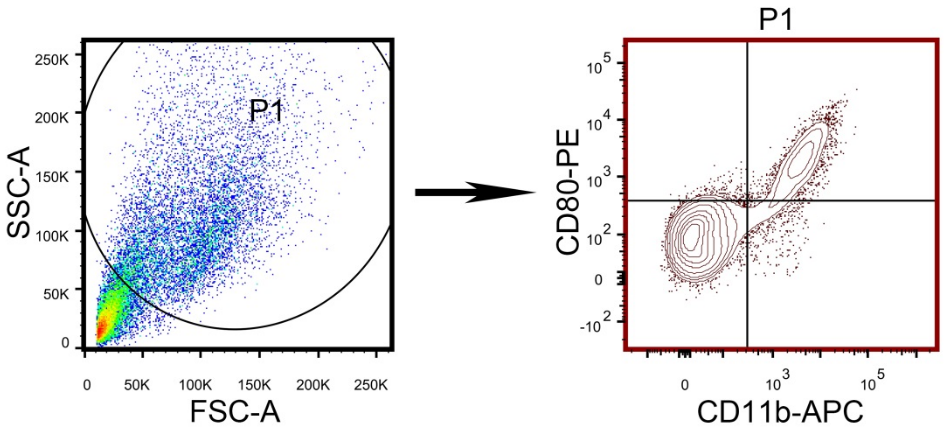

Figure S14. Representative flow cytometric gating strategy for detecting the percentage of M1 macrophages in tumors. Related to Figure 5D.

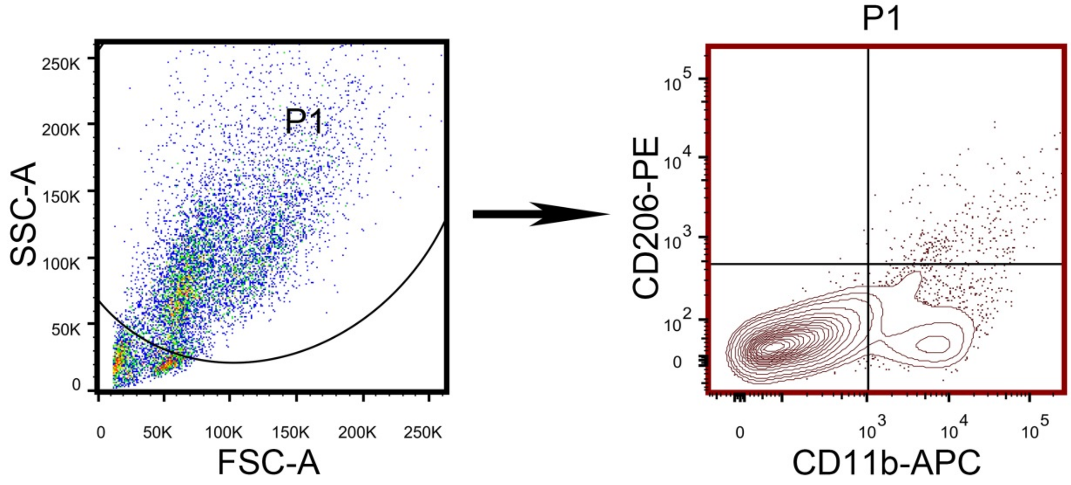

Figure S15. Representative flow cytometric gating strategy for detecting the percentage of M2 macrophages in tumors. Related to Figure 5D.

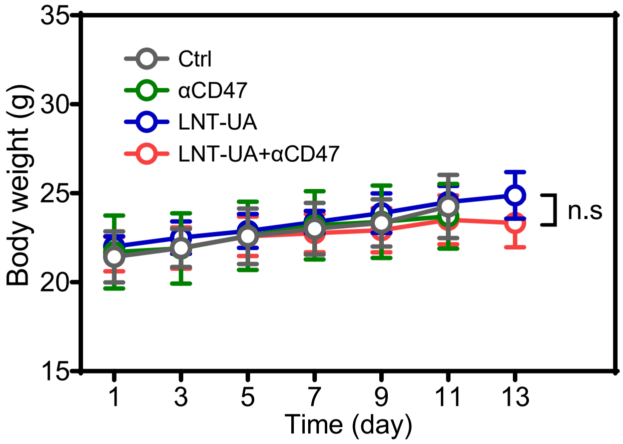

Figure S16. Body weight changes of mice with different treatments in bilateral tumor model. Related to Figure 6.

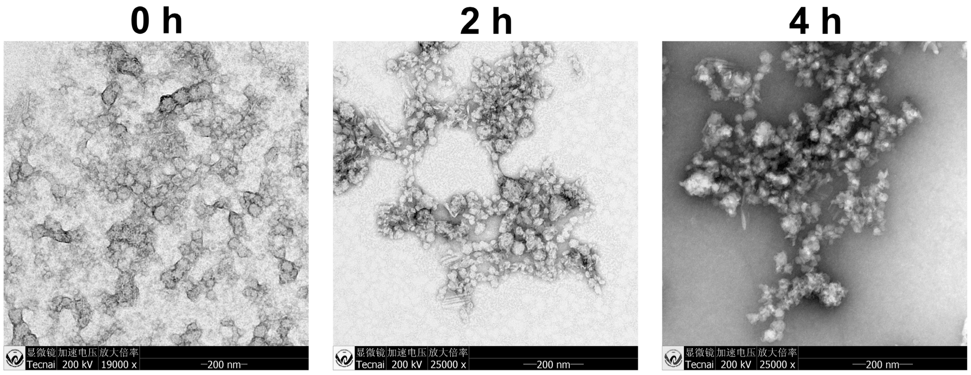

Figure S17. Representative TEM images of LNT-UA storied at pH 2.0 for different times.

Table S1. Particle size and PDI of LNT-UA in water for 7 days.

Time	Size (nm)	PDI
Day 1	74.17±5.64	0.161±0.016
Day 2	77.30±7.24	0.143±0.017
Day 3	75.83±8.09	0.161±0.011
Day 4	73.11±0.61	0.163±0.009
Day 5	67.96±6.20	0.189±0.008
Day 6	71.53±2.51	0.156±0.011
Day 7	76.03±1.52	0.179±0.011
